# Expression of microRNA and their gene targets are dysregulated in preinvasive breast cancer

**DOI:** 10.1186/bcr2839

**Published:** 2011-03-04

**Authors:** Bethany N Hannafon, Paola Sebastiani, Antonio de las Morenas, Jining Lu, Carol L Rosenberg

**Affiliations:** 1 Department of Medicine, Boston Medical Center and Boston University School of Medicine, 72 East Concord Street, Boston, MA 02118, USA; 2 Department of Biostatistics, Boston University School of Public Health, 715 Albany Street, Boston, MA 02118, USA; 3 Department of Pathology, Boston Medical Center and Boston University School of Medicine, 72 East Concord Street, Boston, MA 02118, USA

## Abstract

**Introduction:**

microRNA (miRNA) are short, noncoding RNA that negatively regulate gene expression and may play a causal role in invasive breast cancer. Since many genetic aberrations of invasive disease are detectable in early stages, we hypothesized that miRNA expression dysregulation and the predicted changes in gene expression might also be found in early breast neoplasias.

**Methods:**

Expression profiling of 365 miRNA by real-time quantitative polymerase chain reaction assay was combined with laser capture microdissection to obtain an epithelium-specific miRNA expression signature of normal breast epithelium from reduction mammoplasty (RM) (*n *= 9) and of paired samples of histologically normal epithelium (HN) and ductal carcinoma *in situ *(DCIS) (*n *= 16). To determine how miRNA may control the expression of codysregulated mRNA, we also performed gene expression microarray analysis in the same paired HN and DCIS samples and integrated this with miRNA target prediction. We further validated several target pairs by modulating the expression levels of miRNA in *MCF7 *cells and measured the expression of target mRNA and proteins.

**Results:**

Thirty-five miRNA were aberrantly expressed between RM, HN and DCIS. Twenty-nine miRNA and 420 mRNA were aberrantly expressed between HN and DCIS. Combining these two data sets with miRNA target prediction, we identified two established target pairs (miR-195:*CCND1 *and miR-21:*NFIB*) and tested several novel miRNA:mRNA target pairs. Overexpression of the putative tumor suppressor miR-125b, which is underexpressed in DCIS, repressed the expression of *MEMO1*, which is required for ErbB2-driven cell motility (also a target of miR-125b), and *NRIP1/RIP140*, which modulates the transcriptional activity of the estrogen receptor. Knockdown of the putative oncogenic miRNA miR-182 and miR-183, both highly overexpressed in DCIS, increased the expression of chromobox homolog 7 (*CBX7*) (which regulates E-cadherin expression), *DOK4, NMT2 *and *EGR1*. Augmentation of *CBX7 *by knockdown of miR-182 expression, in turn, positively regulated the expression of E-cadherin, a key protein involved in maintaining normal epithelial cell morphology, which is commonly lost during neoplastic progression.

**Conclusions:**

These data provide the first miRNA expression profile of normal breast epithelium and of preinvasive breast carcinoma. Further, we demonstrate that altered miRNA expression can modulate gene expression changes that characterize these early cancers. We conclude that miRNA dysregulation likely plays a substantial role in early breast cancer development.

## Introduction

Considerable molecular pathology research has focused on invasive breast cancer (IBC); however, less attention has been given to the preinvasive nonobligate precursor, ductal carcinoma *in situ *(DCIS). DCIS is the fourth most common cancer diagnosis among women and is present in the vast majority of IBC cases [1]. Women diagnosed with DCIS are at an increased risk of subsequently developing IBC, and, when examined, DCIS and IBC also share many of the same genetic features. However, there is an increased need to better understand the early genetic events and identify biomarkers that are present prior to IBC. microRNA (miRNA) have emerged as a new class of gene regulators that may serve as both molecular biomarkers and novel therapeutic targets. In this study, we sought to investigate miRNA expression changes and their consequences in preinvasive breast cancer.

miRNA are short, non-protein-coding RNA that exert posttranscriptional control over their mRNA targets through the mechanism of RNA interference. By complementary binding to the 3' untranslated region of target mRNA, miRNA promote mRNA destabilization, thereby inducing translational repression [2]. It has been demonstrated that miRNA control major cellular processes, including metabolism, developmental timing, stem cell division, cell growth and differentiation and apoptosis [3-5]. Given this expansive role, it is unsurprising that their effect on mRNA expression contributes to the pathogenesis of many diseases, including cancer [6,7]. To date, more than 900 miRNA have been identified in humans, constituting more than 1% of the total coding genome. It is predicted that more than 60% of mRNA may be targeted and that a single miRNA may target as many as 200 mRNA, thus making miRNA the largest class of gene regulators [8-10].

Several studies have established the role of miRNA in the pathogenesis of IBC. For example, abnormal miRNA expression has been described in breast cancer cell lines and in bulk primary normal and cancerous breast tissues [11-13]. In this setting, miRNA expression has correlated with specific breast cancer biopathologic features, such as estrogen receptor (ER) and progesterone receptor (PR) expression, tumor stage, vascular invasion or proliferation index. In addition, many miRNA that are consistently downregulated may act as tumor suppressors, for example, miR-206, miR-17-5p, miR-125a, miR-125b and the let-7 family, and many that are consistently upregulated may acts as oncogenes, for example, miR-21, miR-10b and miR-27a. Other studies have shown that miRNA exhibit a specific spatial distribution of expression within breast epithelium [14].

Almost all human breast cancers arise in the epithelial compartment, likely as a result of the transformation of epithelial cells, although the surrounding stroma and microenvironment play a crucial role in tumor progression. Therefore, the present work is focused on the genetic changes that occur within the epithelial cell population.

We hypothesized that miRNA expression might be dysregulated prior to IBC, that these changes might be associated with mRNA expression changes and that together these might help to elucidate important steps in early breast tumorigenesis. Therefore, to first obtain a profile of normal miRNA expression, we profiled miRNA in normal epithelium from healthy controls undergoing reduction mammoplasty (RM). Next, to obtain a profile of miRNA dysregulated prior to invasion, we examined miRNA expression in histologically normal (HN) epithelium and compared this to paired samples of adjacent DCIS. We then integrated the HN:DCIS miRNA expression profile with the gene expression profile from the same samples and used miRNA target prediction programs to identify putative miRNA:mRNA functional interactions. We then selected three candidate miRNA (miR-125b, miR-182 and miR-183) and six of their putative target genes (*MEMO1, NRIP1, CBX7, DOK4, NMT2*, and *EGR1*) for validation. This study represents the first report of a miRNA expression profile in normal breast epithelium and the first integrated analysis of dysregulated miRNA and mRNA expression in paired HN and DCIS samples. Many of the dysregulated miRNA identified in DCIS have previously been identified in IBC. Our data suggest an important role for miRNA in determining the parallel gene expression changes that characterize the earliest stage of breast disease.

## Materials and methods

### Tissue sample acquisition and preparation

Primary breast tissues not needed for diagnosis were obtained at Boston Medical Center from patients undergoing RM and breast cancer surgery (prior to any chemo- or radiation therapy). All samples were deidentified and assigned a number at the time of collection; therefore, informed consent was not required according to our specimen collection protocol preapproved by the Boston University Medical Center Institutional Review Board. Samples were processed as described previously [15]. Epithelia from three groups were examined: normal breast tissue (*n *= 9) from RM (mean age, 52.2 years; age range, 44 to 75 years) and paired samples of HN and DCIS (*n *= 16) from eight individuals undergoing cancer surgery. Hematoxylin and eosin-stained sections were reviewed by a pathologist (AdlM) to verify normal epithelia and preinvasive lesions.

### Laser capture microdissection and RNA isolation

Laser capture microdissection (LCM) was performed as described previously [15-17] to collect breast epithelial cells of normal appearing ductal tissue (RM and HN) and epithelial cells of identified regions of DCIS. Total RNA was isolated using the RNAqueous miRNA Isolation Kit (Ambion, Austin, TX, USA) and treated with DNase I according to the manufacturer's instructions. RNA to be utilized for gene expression analysis was processed as described previously [15,16]. The pooled RM sample was prepared by combining 400 ng of total RNA from each of the nine RM samples.

### miRNA expression profiling and statistical analysis

cDNA was synthesized from 800 ng (100 ng/multiplex pool) with the TaqMan miRNA Reverse Transcription Kit (Applied Biosystems, Foster City, CA, USA), according to manufacturer's instructions. miRNA expression was measured by real-time quantitative polymerase chain reaction (RT-qPCR) assay utilizing the TaqMan Human miRNA Array Panel (version 1.0, based on miRBase version 9.2; Applied Biosystems) and assayed on the 7900 Real-Time PCR System (Applied Biosystems), according to the manufacturer's instructions. miRNA expression data are available from the National Center for Biotechnology Gene Expression Omnibus (GEO) [18] at accession number [GEO:GSE24509].

All probes with threshold cycles (Ct) = 40 in more than two of three pooled RM (PRM) replicates or more than six of eight HN samples and more than six of eight DCIS samples were considered "nonexpressed" and removed. Remaining Ct values were global median normalized by transforming all expression values by rescaling to a target value of 12 (ΔCt). Relative changes in miRNA expression among each comparison (HN-PRM, DCIS-PRM and DCIS-HN) were assessed (ΔΔCt ). A variance correction was applied to account for the pooled samples as suggested by Churchill [19], and a *t*-test was performed. *P *< 0.005 in at least one of the comparisons was considered statistically significant. To address the issue of multiple comparisons, we highlight the results that remain significant using two valid procedures: the more restrictive Bonferroni correction (*P *< 0.00025) and the less restrictive false discovery rate < 0.05 (*P *< 0.017), which typically results in a greater number of significant results. The relative fold change for each comparison was calculated by 2^^-ΔΔCt^. Heatmaps were generated using the Heatplus package in Bioconductor [20].

### Gene expression profiling and statistical analysis

Gene expression analysis was measured on the U133A GeneChip (Affymetrix, Santa Clara, CA, USA). All microarray analyses were performed at the Boston University Microarray Facility as previously described [15]. The paired data were assembled as follows: 12 paired samples (six HN and six DCIS) were pulled from the data published by Emery *et al*. [15], two HN samples were pulled from the data published by Graham *et al*. [21], and the two matching pairs of DCIS samples (combined to equal 16 paired samples) were collected and processed from tissue acquired from the same patient. Array data were analyzed as previously described [15]. Microarray output data were filtered by removing all probe sets present in < 15% of all samples. Next, data were analyzed by performing Bayesian Analysis of Differential Gene Expression (BADGE) as previously described by Emery *et al*. [15] and found online at the BADGE website [22]. The gene expression data are available from GEO under accession number [GEO:GSE24509].

### miRNA target prediction

SigTerms [23] was utilized to extract predictions from PicTar [24], TargetScan (4.1 and 5.1) [25] and miRanda (Jan 08 and Sep 08) [26]. miRNA target predictions were extracted two separate times, the first using TargetScan release 4.1 and miRanda release Jan 08 and the second using TargetScan release 5.1 and miRanda release Sep 08. The final prediction results are a combination of the two queries. Pearson correlations and associated *P *values were calculated across all 16 HN and DCIS samples for each of the target pairs identified from the intersection of the programs.

### Gene ontology and pathway analysis

Gene annotation, ontology and pathway analysis were conducted using the Database for Annotation, Visualization and Integrated Discovery [27]. A modified Fisher's exact test/EASE (Enrichment) Score was utilized to calculate the *P*-values.

### miRNA pre-miR and anti-miR transient transfection

MCF7 cells were kindly provided by G. Sonenshein (Tufts-New England Medical Center, Boston, MA, USA) and were maintained in Dulbecco's modified Eagle's medium (Invitrogen, Carlsbad, CA, USA) with 4.5 g/l glucose and sodium pyruvate supplemented with 5.8 g/l L-glutamine (Cellgro, Manassas, VA, USA), 10% fetal bovine serum (Sigma-Aldrich, St. Louis, MO, USA) and 1% penicillin-streptomycin (Cellgro). For all experiments, 5 × 10^4 ^cells/well of a 12-well plate were seeded for 24 hours and then transfected with (1) 100 nM pre-miR-125b or scrambled negative control sequence (Scramble) or (2) 50 nM Anti-miR-182, Anti-miR-183 or Scramble using the siPORT NeoFX Transfection Agent (Ambion).

### RNA extraction cell culture

Cells were rinsed with 1× phosphate-buffered saline (PBS) and lysed with 600 μl of lysis buffer, and total RNA was isolated with the mirVana Isolation Kit (Ambion) and treated with DNase I, according to the manufacturer's instructions. RNA quantity was determined using Quant-it RiboGreen RNA Quantitation Reagent (Invitrogen) according to the manufacturer's instructions.

### qRT-PCR for target gene expression

cDNA from 500 ng of total RNA was synthesized using TaqMan RT reagents according to the manufacturer's instructions. qRT-PCR was performed by diluting RT product in 2× Universal PCR MasterMix and 20× TaqMan Gene Expression Assay for each gene to be measured: chromobox homolog 7 (*CBX7*) (Hs00980916_g1), docking protein 4 (*DOK4*) (Hs00902919_g1), early growth response 1 (*EGR1*) (Hs00152928_m1), glyceraldehyde 3-phosphate dehydrogenase (*GAPDH*) (4333764F), mediator of ErbB2-driven cell motility (*MEMO1*) (Hs00831646_uH), N-myristoyltransferase 2 (*NMT2*) (Hs01013924_g1) and nuclear receptor-interacting protein 1 (*NRIP1/RIP140*) (Hs00942766_s1). PCR reactions were run on the 7500 real-time PCR instrument under the following conditions: hold at 95°C for 10 minutes, then 40 cycles of 95°C for 15 seconds and 60°C for 1 minute. All reagents were purchased from Applied Biosystems. Relative gene expression was assessed using the differences in normalized Ct (ΔΔCt) method after normalization to GAPDH. Fold changes were calculated by 2^^-ΔΔCt^.

### Immunoblot analysis

Cells were washed with 1× PBS 48 hours posttransfection and collected in radioimmunoprecipitation assay buffer (25 mM Tris-HCl, pH 7.6, 150 mM NaCl, 1% NP-40, 1% sodium deoxycholate, 0.1% sodium dodecyl sulfate). Protein (50 μg) was electrophoresed through a 4% to 15% Tris·HCl Ready Gel (Bio-Rad, Hercules, CA, USA) under reducing conditions and transferred onto a polyvinylidene fluoride membrane. The membrane was incubated with primary antibodies against *CBX7 *(ab21873; Abcam, Cambridge, MA, USA), E-cadherin (610181; BD Biosciences, San Jose, CA, USA) and β-actin (A5441; Sigma-Aldrich). Immune complexes were detected using horseradish peroxidase-conjugated secondary antibodies and the SuperSignal West Pico Chemiluminescent Substrate Kit (Pierce Biotechnology, Rockford, IL, USA).

## Results

### miRNA expression profiles of RM, HN and paired DCIS

Our first goal was to generate a set of miRNA expression profiles in primary human healthy and diseased breast epithelia. Therefore, we microdissected 25 samples to enrich for epithelial RNA from 17 patients (see Additional file 1 for representative epithelial lesions) separated into three groups: the control group of normal epithelia from nine patients undergoing RM and the paired diseased groups, which consisted of 16 samples from eight patients (eight samples of HN and eight samples of adjacent ER- and PR-positive DCIS (Table 1).

**Table 1:** Paired histologically normal and DCIS sample summary^a^

Sample	Age, yr	DCIS grade	DCIS histology	ER/PR/HER2 of IDC	IDC grade
379	43	2 and 3	Cribiform	+/+/+	2
444	48	2	Cribiform	+/+/NA	Not present
248	49	1	Micropapillary	+/+/NA	2
274	49	2	Solid	+/+/-	1
380	53	2 and 3	Solid/cribiform	+/+/-	2
446B	54	3	Comedo/solid	+/+/-	2
258	65	1 and 2	Cribiform/micropapillary	+/+/-	2
405	67	2	Micropapillary/cribiform	+/+/-	2
Mean age	53.5				
Median age	51.0				

^a^DCIS, ductal carcinoma *in situ*; ER, estrogen receptor; PR, progesterone receptor; HER2, human epidermal growth factor receptor 2; IDC, invasive ductal carcinoma *in situ*; NA, not applicable.

Next, to test the reproducibility of the miRNA expression array, we combined equal amounts of RNA from nine RM samples into a pooled RM (PRM). The PRM served both as a heterogeneous biological control and as a technical replicate. The PRM was run in triplicate and showed a high correlation between each replicate with a mean Pearson's correlation of 0.95 (Additional file 2).

### miRNA are differentially expressed in normal and preinvasive breast cancer

**Figure 1:**
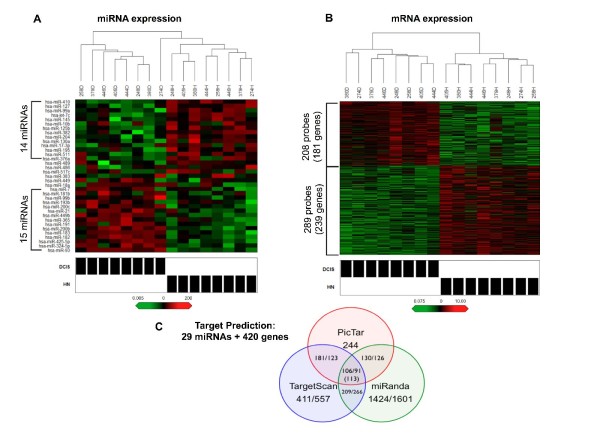
**Expression profiling and target prediction of microRNA (miRNA) and genes in histologically normal (HN) and ductal carcinoma *in situ *(DCIS)**. **(A) **and **(B) **Hierarchical clustering heatmaps of **(A) **miRNA (*P *< 0.005, fold change >3) and **(B) **mRNA (*P *< 0.05, fold change >1.5) overexpressed (red) and underexpressed (green) in DCIS versus paired HN. Brackets indicate the most significant miRNA and probe sets. Rows, transcripts/miRNA; columns, profiled patient samples; shaded boxes, lesion type. **(C) **Venn diagram depicting number of predicted functional pairs for each miRNA target prediction algorithm (PicTar, TargetScan and miRANDA) and the intersection of algorithms, first and second queries are separated by a forward slash.

By comparing the miRNA expression profiles between PRM, HN and DCIS (see Additional file 3 for a list of miRNA expressed in each group), we found that 35 miRNA were differentially expressed (*P *< 0.005) in at least one comparison (see heatmaps in Figure 1A and Additional file 4, as well as data in Table 2). As expected, the fewest differences were found in the HN-PRM comparison, where 11 miRNA were different (seven overexpressed and four underexpressed). More than twice that number were different in both the DCIS-PRM and DCIS-HN comparisons, with 29 miRNA being different and 23 of 29 overlapping between these comparisons. In the DCIS-PRM comparison, 17 miRNA were overexpressed and 12 were underexpressed; in the DCIS-HN comparison, 15 miRNA were overexpressed and 14 were underexpressed. It has been noted that miRNA that are present within 50 kb at the same genetic loci are often coordinately expressed [28]. We examined these 35 miRNA for coordinate expression because of their close genomic proximity. The 35 miRNA were located at 29 different loci, and 16 of 35 miRNA were clustered at seven distinct loci (indicated in Additional file 4). As expected, 14 (88%) of 16 miRNA < 13 kb apart were positively correlated, with the exception of miR-17-3p and miR-18a.

**Table 2:** Differentially expressed microRNA among pooled reduction mammoplasty, histologically normal and ductal carcinoma *in situ*^a^

	HN versus PRM		DCIS versus PRM		DCIS versus HN	
			
**microRNA**	Fold change	*P *value^b^	Fold change	*P *value^b^	Fold change	*P *value^b^
let-7c [62]	15.23	3.46E-03^c^	0.31	1.25E-01	0.03	1.19E-03^c^
miR-7 [38]	3.12	1.35E-01	22.35	1.53E-03^c^	5.19	4.92E-02
miR-10b [13,39]	2.90	1.59E-01	0.82	7.82E-01	0.04	2.58E-03^c^
miR-17-3p [32,40]	19.04	2.14E-03^c^	2.20	2.85E-01	0.12	1.73E-02
miR-18a [34]	1.91	3.76E-01	20.88	1.76E-03^c^	3.53	1.12E-01
miR-21 [13,31,36,40,41]	6.74	2.23E-02	55.20	2.64E-04^c^	20.92	3.21E-03^c^
miR-93[34]	7.53	1.72E-02	193.86	3.33E-05d	160.47	1.59E-04d
miR-99a	1.90	3.79E-01	0.53	3.79E-01	0.02	9.19E-04^c^
miR-99b	3.08	1.39E-01	14.84	3.66E-03^c^	6.24	3.33E-02
miR-125b [13,29]	0.93	9.24E-01	0.08	5.39E-03^c^	0.05	4.03E-03^c^
miR-127 [30,31]	1.77	4.30E-01	0.25	7.81E-02	0.02	5.30E-04^c^
miR-130a	0.29	1.06E-01	0.01	1.38E-04^d^	0.09	1.01E-02
miR-145 [13,14]	3.00	1.47E-01	0.80	7.52E-01	0.03	1.20E-03^c^
miR-181b [31,40]	3.40	1.11E-01	14.90	3.64E-03^c^	5.46	4.42E-02
miR-182 [33]	2.00	3.45E-01	106.24	8.54E-05^d^	72.35	4.55E-04^c^
miR-183	2.52	2.15E-01	19.06	2.14E-03^c^	51.88	7.38E-04^c^
miR-191 [13]	6.75	2.23E-02	78.21	1.43E-04^d^	31.59	1.60E-03^c^
miR-193b [18]	2.20	2.84E-01	15.67	3.25E-03^c^	6.87	2.73E-02
miR-195 [35]	1.37	6.57E-01	0.06	2.78E-03^c^	0.13	2.15E-02
miR-200b [37]	1.70	4.63E-01	6.07	2.86E-02	51.31	7.50E-04^c^
miR-200c [37]	1.90	3.80E-01	26.41	1.08E-03^c^	19.98	3.48E-03^c^
miR-204 [13]	0.77	7.12E-01	0.04	1.32E-03^c^	0.08	8.12E-03^c^
miR-324-5p	2.86	1.64E-01	24.38	1.28E-03^c^	92.53	3.24E-04^c^
miR-365 [31]	4.05	7.45E-02	17.65	2.51E-03^c^	25.05	2.35E-03^c^
miR-376a	0.45	2.73E-01	0.06	3.14E-03^c^	0.24	7.91E-02^c^
miR-382	2.03	3.32E-01	0.11	1.20E-02^c^	0.06	4.71E-03^c^
miR-383 [34]	0.05	2.15E-03^c^	0.01	1.58E-04^d^	0.62	5.18E-01
miR-410	0.96	9.56E-01	0.15	2.33E-02^c^	0.00	1.11E-04^d^
miR-425-5p	1.38	6.57E-01	9.47	1.01E-02^c^	74.46	4.37E-04^c^
miR-449a	10.65	7.71E-03^c^	15.12	3.52E-03^c^	2.53	2.23E-01
miR-449b	0.79	7.37E-01	11.57	6.39E-03^c^	21.20	3.13E-03^c^
miR-486	0.02	2.06E-04^d^	0.01	9.73E-05^d^	0.34	1.65E-01
miR-489	0.06	2.73E-03^c^	0.10	8.61E-03^c^	0.25	8.62E-02
miR-511	0.40	2.20E-01	0.07	3.72E-03^c^	0.18	4.11E-02
miR-517^c^	0.07	3.48E-03^c^	0.01	1.29E-04^d^	0.36	1.82E-01

^a^RM, reduction mammoplasty; PRM, pooled reduction mammoplasty; HN, histologically normal; DCIS, ductal carcinoma *in situ*; microRNA in boldface type have previously been implicated in invasive breast cancer; ^b^*P *value determined by Student's *t*-test; ^c^*P *< 1.7E-02 implies significance with a false-discovery rate of 0.05; ^d^*P *< 2.5E-04 implies significance after the Bonferroni correction; fold change in expression between comparisons was considered significant if *P *< 5E-02.

To validate our miRNA data set, we searched the literature to determine whether any of these miRNA are implicated in IBC. Twenty (57%) of the 35 miRNA have previously been implicated (noted in Table 2) [13,14,18,29-41]. Eleven of these 20 miRNA were overexpressed in DCIS compared to HN and PRM (miR-181b, miR-200b, miR-200c, miR-18a, miR-21, miR-365, miR-7, miR-182, miR-191, miR-193b and miR-93). The directional change in expression in DCIS of seven (64%) of these 11 miRNA is consistent with their reported expression in IBC; the exceptions are miR-18a, miR-193b, miR-200b and miR-200c. Nine of these 20 miRNA were underexpressed in DCIS compared to HN and PRM (let-7c, miR-10b [14], miR-125b, miR-127, miR-145, miR-17-3p, miR-195, miR-204 and miR-383), and the directional change in expression in DCIS of eight (89%) of these nine miRNA is consistent with their expression in IBC (except miR-195). Overall, 15 (75%) of 20 miRNA that are dysregulated are concordantly dysregulated in IBC. Of the 29 miRNA dysregulated in DCIS compared to HN, 18 (62%) of 29 are implicated in IBC and concordant with the reported changes in expression.

### Differentially expressed miRNA are predicted to target differentially expressed genes in HN-DCIS comparison

The identification of miRNA targets is crucial to understanding the biological role of miRNA. We have recently shown that gene expression is altered in paired HN and DCIS epithelial cells [15]. Because mRNA destabilization is a mechanism of miRNA-mediated gene repression [39], we sought to determine whether any of the 29 miRNA altered in the HN-DCIS comparison may participate in the regulation of these differentially expressed genes through this mechanism. First, we examined the gene expression profile from the same 16 paired HN and DCIS samples. Data were analyzed by BADGE. BADGE uses a model-averaging approach to calculate the posterior probability of a fold change > 1 for each probe set and ranks the genes, so that probe sets with a very small probability (< 0.025) or a very large one (> 0.975) are considered differentially expressed. On the basis of the BADGE analysis, we selected the probe sets in which the probability of a fold change > 1.5 was either > 0.975 or < 0.025. These probe sets were further analyzed using a linear mixed model with lognormal errors and random effects to account for patient matching across probe sets. On the basis of this analysis, we obtained a set of 497 probe sets (420 genes) that were differentially expressed (*P *< 0.05, fold change > 1.5) between HN and DCIS, 208 probe sets (181 genes) that were overexpressed and 289 probe sets (239 genes) that were underexpressed (Figure 1B). The list of probe sets and mean expression values is provided in Additional file 5.

We then combined the two expression profiles to identify putative miRNA:mRNA functional pairs linking these 420 mRNA and 29 miRNA. To increase specificity (at the cost of lower sensitivity), we integrated results of three target prediction programs and examined only the intersection. We found that 113 unique miRNA:mRNA target pairs were predicted by all three programs, composed of 74 genes and 13 miRNA (Figure 1C).

Returning to the expression data, we found that 59 of 113 miRNA:mRNA pairs (45 mRNA and 12 miRNA) were inversely expressed and that 54 of 113 miRNA:mRNA pairs (46 mRNA and 13 miRNA) were coordinately expressed. The inverse pairs are the canonical understanding of miRNA:mRNA interactions, meaning that as the expression of one changes, it is expected that the expression of the other will change in the opposite direction. The coordinately expressed pairs can represent either false-positive predictions or positive target regulation. The degree of anticorrelation for the inverse pairs was calculated, and the results are listed in Table 3. Similarly, the degree of correlation for the coordinate pairs was calculated, and the data are provided in Additional file 6.

**Table 3:** Expression correlation of microRNA and inversely expressed predicted targets^a^

microRNA expression		Gene expression			miRNA:mRNA	
**miRNA**	**Fold change**	**Probe ID**	**Gene symbols, gene names**	**Fold change**	**Expression correlation**	***P *value^b^**

let-7^c^	0.03	203481_at	*C10ORF6*, chromosome 10 open reading frame 6	1.58	-0.58	1.88E-02
		218567_x_at	*DPP3*, dipeptidylpeptidase 3	2.31	-0.49	5.4E-02
		203358_s_at	*EZH2*, enhancer of zeste homolog 2 (*Drosophila*)	2.36	-0.64	7.69E-03
		209283_at	*SLC20A1*, solute carrier family 20 (phosphate transporter), member 1	2.06	-0.30	2.53E-01
		203358_s_at	*TRIB1*, tribbles homolog 1 (*Drosophila*)	1.77	-0.48	5.84E-02

miR-10b	0.04	202357_s_at	*SDC1*, syndecan 1	2.06	-0.28	2.96E-01

miR-125b	0.05	203744_at	*HMGB3*, high-mobility group box 3	2.13	-0.61	1.27E-02
		**209613_s_at**	**NRIP1, nuclear receptor interacting protein 1**	**2.25**	**-0.39**	**1.36E-01**
		**213004_at**	**MEMO1, mediator of cell motility 1**	**1.73**	**-0.59**	**1.54E-02**

miR-17-3p	0.12	203744_at	*HMGB3*, high-mobility group box 3	2.13	-0.49	5.66E-02
		212446_s_at	*LASS6*, LAG1 longevity assurance homolog 6	1.78	-0.31	2.38E-01
		211653_x_at	*MAP7*, microtubule-associated protein 7	1.73	-0.49	5.19E-02
		213492_at	*SERP1*, stress-associated endoplasmic reticulum protein 1	1.80	-0.23	3.84E-01
		203213_at	*SAR1B*, SAR1 gene homolog B (Saccharomyces cerevisiae)	1.95	-0.07	8.03E-01
		202381_at	*ADAM9*, ADAM metallopeptidase domain 9	2.04	-0.45	7.89E-02

miR-181b	5.46	221234_s_at	*BACH2*, BTB and CNC homology 1 basic leucine zipper transcription factor 2	0.47	-0.30	2.62E-01
		212914_at	*CBX7*, chromobox homolog 7	0.44	-0.30	2.67E-01
		
		204753_s_at 204755_x_at 204754_at	*HLF*, hepatic leukemia factor	0.24 0.28 0.31	-0.19 -0.52 -0.45	4.84E-01 3.82E-02 7.70E-02
		
		204567_s_at	*NMT2*, N-myristoyltransferase 2	0.58	-0.35	1.81E-01
		202274_at 200974_at	*NR3C1*, nuclear receptor subfamily 3, group C, member 1 (glucocorticoid receptor)	0.46 0.57	-0.44 -0.57	8.86E-02 2.21E-02

miR-182	72.35	221234_s_at	*BACH2*, BTB and CNC homology 1 basic leucine zipper transcription factor 2	0.47	-0.73	1.41E-03
		210347_s_at 219497_s_at 219498_s_at	*BCL11A*, B-cell CLL/lymphoma 11A	0.27 0.42 0.43	-0.65 -0.69 -0.80	6.31E-03 2.86E-03 2.19E-04
		221530_s_at	*BHLHB3*, basic helix-loop-helix domain containing, class B, 3	0.47	-0.55	2.71E-02
		204851_s_at 204850_s_at	*DCX*, doublecortex; lissencephaly, X-linked (doublecortin)	0.13 0.30	-0.83 -0.67	7.48E-05 4.63E-03
		**209691_s_at**	**DOK4, docking protein 4**	**0.56**	**-0.53**	**3.65E-02**
		
		209905_at 214651_s_at	*HOXA9*, homeobox A9	0.10 0.31	-0.64 -0.55	8.18E-03 2.63E-02
		
		64900_at	*LPHN2*, latrophilin 2	0.37	-0.62	9.87E-03
		219497_s_at	*PRKD1*, protein kinase D1	0.54	-0.59	1.61E-02
		210735_s_at	*RIMS3*, regulating synaptic membrane exocytosis 3	0.52	-0.73	1.25E-03
		205022_s_at	*FOXN3*, checkpoint repressor 1	0.50	-0.67	4.40E-03
		221935_s_at	*NCAM1*, neural cell adhesion molecule 1	0.43	-0.41	1.19E-01
		**212914_at**	**CBX7, chromobox homolog 7**	**0.44**	**-0.57**	**2.22E-02**
		**204567_s_at**	**NMT2, N-myristoyltransferase 2**	**0.58**	**-0.26**	**3.26E-01**

miR-183	51.88	221234_s_at	*BACH2*, BTB and CNC homology 1 basic leucine zipper transcription factor 2	0.47	-0.75	7.83E-04
		
		204851_s_at 204850_s_at	*DCX*, doublecortex; lissencephaly, X-linked (doublecortin)	0.13 0.30	-0.82 -0.80	1.06E-04 2.16E-04
		
		**201693_s_at**	**EGR1, early growth response 1**	**0.45**	**-0.61**	**1.12E-02**
		202274_at 200974_at	*NR3C1*, nuclear receptor subfamily 3, group C, member 1 (glucocorticoid receptor)	0.46 0.57	-0.70 -0.45	2.77E-03 8.08E-02

miR-195	0.13	217852_s_at	*ARL8B*, ADP ribosylation factor-like 8B	1.64	-0.31	2.49E-01
		208712_at	***CCND1, cyclin D1 *** [43]	2.30	-0.33	2.19E-01
		208653_s_at	*CD164*, CD164 molecule, sialomucin	2.40	-0.54	3.16E-02
		202596_at	*ENSA*, endosulfine α	2.03	-0.37	1.54E-01
		211653_x_at	*MAP7*, microtubule-associated protein 7	1.73	-0.52	4.00E-02
		221935_s_at	*RAD23B*, RAD23 homolog B	1.79	-0.08	7.61E-01
		201341_at	*TMEM33*, transmembrane protein 33	1.62	-0.30	2.64E-01
		
		212464_s_at 216442_x_at	*WWP1*, WW domain containing E3 ubiquitin protein ligase 1	2.79 2.83	-0.49 -0.25	5.57E-02 5.57E-02

miR-204	0.08	217852_s_at	*ARL8B*, ADP-ribosylation factor-like 8B	1.64	-0.27	3.06E-01
		213492_at	*SERP1*, stress-associated endoplasmic reticulum protein 1	1.80	-0.57	2.22E-02

miR-21	20.92	218992_at 219060_at	***NFIB, nuclear factor I/B *** [42]	0.43 0.52	-0.75 -0.69	9.15E-04 3.28E-03

miR-7	5.19	213492_at	*COL2A1*, collagen type II, α1	0.51	-0.12	6.61E-01
		204359_at	*FLRT2*, fibronectin leucine-rich transmembrane protein 2	0.35	-0.49	5.36E-02
		206765_at	*KCNJ2*, potassium inwardly rectifying channel, subfamily J, member 2	0.33	-0.73	1.35E-03
		214112_s_at	*SNCA*, synuclein α	0.48	-0.54	3.00E-02
		205022_s_at	*FOXN3*, checkpoint repressor 1	0.50	-0.61	1.22E-02

miR-93	160.47	210347_s_at 219497_s_at 219498_s_at	*BCL11A*, B-cell CLL/lymphoma 11A	0.27 0.42 0.43	-0.75 -0.72 -0.71	7.40E-04 1.51E-03 2.11E-03
		
		204753_s_at 204755_x_at 204754_at	*HLF*, hepatic leukemia factor	0.24 0.28 0.31	-0.68 -0.81 -0.81	3.60E-03 1.44E-04 1.49E-04

		218992_at 219060_at	*NFIB*, nuclear factor I/B	0.43 0.52	-0.74 -0.65	1.14E-03 6.30E-03
		
		203963_at	*RGL1*, ral guanine nucleotide dissociation stimulator-like 1	0.53	-0.58	1.79E-02
		
		204422_s_at 205117_at	*TXNIP*, thioredoxin-interacting protein	0.36 0.49	-0.69 -0.57	2.96E-03 2.14E-02

^a^miRNA, microRNA; miRNA:target mRNA pairs tested in this study are indicated by boldface type, and previously confirmed targets from the literature are indicated by boldface italicized type, ^b^*P *value determined by Student's *t*-test.

### Gene ontology and pathway analysis of gene targets reveals enriched involvement in transcription

To determine whether a particular molecular or biological function or pathway was overrepresented among the 74 predicted target genes, we conducted gene ontology and pathway analysis. According to these results, 23 (31%) of 74 of the genes regulate transcription. Of the 23, 15 have transcription factor activity, four are transcriptional repressors and nine are sequence-specific DNA-binding factors (see Additional file 7). For example, *EGR1 *and homeobox A9 (*HOXA9*) are predicted targets (miR-183:*EGR1 *and let-7c/miR-182:*HOXA9*) and are both transcription factors that regulate many genes, including eukaryotic translation initiation factor 4E (*EIF4E*), collagen type II, α1 (*COL2A1*), *NAB1 *and *SNAIL *(by *EGR1*), as well as *EIF4E *and *MEIS2 *(by *HOXA9*). Interestingly, these genes were also differentially expressed in the HN-DCIS comparison, and several of these genes are also predicted targets (miR-7:*COL2A1*, let-7c:*NAB1 *and let-7c:*MEIS2*). Because of the use of such a small gene list, no pathways reached a level of significance (see Additional file 8); however, we noted that several genes are associated with cancer-related pathways, such as cell cycle regulation (cyclin D1 (*CCND1*) and *YWHAZ*), mitogen-activated protein kinase (MAPK) signaling (fibroblast growth factor 2 (*FGF2*) and *DOK4*), nucleotide excision repair (RAD23 homolog B (*RAD23B*)) and *p53 *interactions (*CCND1, CBX7*, enhancer of zeste homolog 2 (*Drosophila*) (*EZH2*), WW domain containing E3 ubiquitin protein ligase 1 (*WWP1*) and *PTB4A1*).

### miR-125b, miR-182 and miR-183 target validation

Two of the 59 inverse miRNA:mRNA target pairs we identified have been validated: miR-21:*NFIB *(an oncogenic interaction) in leukemia cells [42] and miR-195:*CCND1 *(a tumor-suppressive interaction) in hepatocellular carcinoma [43]. Our data suggest a new role for these proven interactions in DCIS.

We wished to validate additional miRNA:mRNA inverse target pairs. On the basis of a high degree of differential expression in DCIS, multiple predicted targets and potential relevance to cancer, we selected six of 59 inverse target pairs consisting of three miRNA (miR-125b, miR-182 and miR-183) for experimental manipulation in a breast cancer cell line.

miR-125b expression is greatly reduced in DCIS compared to HN (0.05-fold) and PRM (0.08-fold). Target prediction analysis identified three inverse putative miR-125b targets (Table 3). We selected *MEMO1 *and *NRIP1 *for validation. *MEMO1 *is a nonheme iron-dependent dioxygenase that binds to the C-terminus of ErbB2/Her2 (also a known target of miR-125b) and is required for ErbB2-driven cell motility [29,44]. *NRIP1/RIP140 *is a nuclear protein that modulates the transcriptional activity of the ER [45].

**Figure 2:**
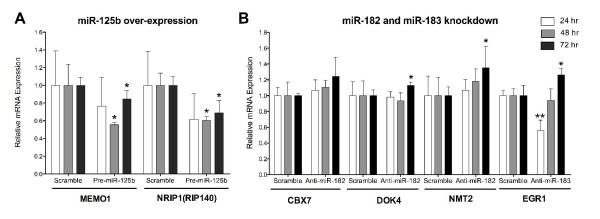
**Effect of miR-125b overexpression and miR-182 and miR-183 knockdown on predicted target gene expression**. **(A) **Reduced average relative fold change in expression of predicted miR-125b targets: mediator of ErbB2-driven cell motility 1 (*MEMO1*) (*P < 0.08) and nuclear receptor interacting protein 1 (*NRIP1(RIP140)*) (*P < 0.05). **(B) **Increased average relative fold change in expression of predicted miR-182 targets: chromobox homolog 7 (*CBX7*), docking protein 4 (*DOK4*) and N-myristoyltransferase 2 (*NMT2*) and miR-183 target early growth response 1 (*EGR1*) (*P < 0.05 and **P < 0.02). Target mRNA expression was measured at 24, 48 and 72 hours posttransfection (*n *= 4, all conditions). Significant differences were determined by Student's *t*-test. Error bars indicate standard error of the mean.

To determine whether *MEMO1 *and *NRIP1 *are authentic miR-125b targets, we transiently expressed the precursor of miR-125b in MCF7 cells. This reduced the endogenous expression of *MEMO1 *as early as 24 hours (0.75-fold), and expression was significantly further decreased at 48 hours (0.62-fold) and remained below baseline at 72 hours (0.84-fold). Similarly, the expression of *NRIP1 *was reduced at 24 hours (0.63-fold), and expression was significantly further decreased at 48 hours (0.53-fold) and remained below baseline at 72 hours (0.68-fold) (Figure 2A). These results suggest that both *MEMO1 *and *NRIP1 *are negatively regulated by miR-125b.

miR-182 and miR-183 expression is greatly increased in DCIS compared to HN (72.35- and 51.88-fold, respectively) and to PRM (106.24- and 19.06-fold, respectively), and they are slightly increased in HN compared to PRM (2.0- and 2.52-fold, respectively). Target prediction analysis identified 13 inverse targets of miR-182 and four inverse targets of miR-183 (Table 3). We selected *CBX7, DOK4 *and *NMT2 *for miR-182 validation and *EGR1 *for miR-183 validation. *CBX7 *is a chromobox family protein and a member of the polycomb-repressive complex 1 that positively regulates E-cadherin expression through interaction with *HDAC2 *[46]. *DOK4 *acts as an anchor for c-Src kinase, inhibits tyrosine kinase signaling and can activate MAPK [47,48]. *NMT2 *is a N-myristoyltransferase, and myristoylated proteins have diverse biological functions in signal transduction and oncogenesis [49]. *EGR1 *is a Cys_2_His_2_-type zinc finger protein that functions as a transcriptional regulator of target genes required for differentiation and mitogenesis [50].

To determine whether these genes are authentic targets of miR-182 and miR-183, we introduced an antisense RNA specifically designed to knock down the expression of mature miR-182 and miR-183 into MCF7 cells. We observed that from 24 through 72 hours, there was an upward trend in expression for each of the target genes, which was significant for three of the four targets at 72 hours. By 72 hours, the increases in endogenous expression were 1.23-fold for *CBX7*, 1.13-fold for *DOK4 *and 1.34-fold for *NMT2. EGR1 *was unexpectedly decreased at 24 hours (0.46-fold), but its expression had returned to baseline at 48 hours and was increased at 72 hours (1.2-fold) (Figure 2B).

### Knockdown of miR-182 induces the expression of E-cadherin through upregulation of *CBX7*

**Figure 3:**
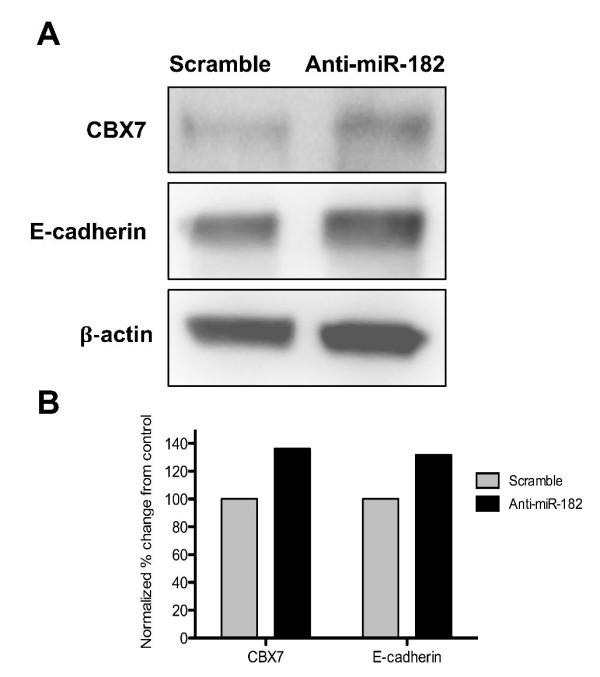
**Effect of miR-182 on chromobox homolog 7 (*CBX7*) and E-cadherin expression**. **(A) **Protein levels of *CBX7 *and E-cadherin after transfection with anti-miR-182 or scramble. β-actin served as a loading control. **(B) **The percentage changes in levels of *CBX7 *and E-cadherin were quantitated and normalized for β-actin.

It has been suggested that the loss of *CBX7 *expression may influence the invasiveness of epithelial cancers by promoting an epithelial-to-mesenchymal transition [46]. This effect is believed to be due to *CBX7*'s ability to promote the expression of E-cadherin, a cell adhesion molecule that plays a role in maintaining normal epithelial cell morphology by associating with and inhibiting the repressive action of *HDAC2 *within the E-cadherin promoter region. Loss of E-cadherin expression during neoplastic progression is associated with several cancers, including breast cancer. In this study, the expression of *CBX7 *was reduced 0.44-fold in the DCIS-HN comparison; however, we did not observe a significant decrease of E-cadherin in our preinvasive clinical samples. It may be that the loss of E-cadherin expression is more characteristic of the invasive transition or of lobular histology. However, we asked whether upregulation of *CBX7 *due to the reduction of its targeting miRNA (miR-182) would also lead to upregulation of E-cadherin expression *in vitro*. We found that by 48 hours postknockdown of miR-182, the protein levels of both *CBX7 *and E-cadherin were upregulated by approximately 35% to 40% relative to control (Figures 3A and 3B).

## Discussion

In this study, we identified a set of miRNA that are expressed in normal breast epithelium and found that major miRNA expression changes occur at the transition from normal to DCIS epithelium, thereby defining a set of putative oncogenic and tumor suppressor miRNA that are dysregulated at the preinvasive stage of breast cancer. A greater number of miRNA were expressed in PRM compared to HN and DCIS, which is contrary to what we have observed in our gene expression studies, and we recognize that the high C_t _cutoff values employed could have influenced these results [15,16]. However, this observation fits with the current understanding of miRNA regulation of mRNA expression, given that a greater number of expressed miRNA would correspond to fewer expressed mRNA.

Twenty of these miRNA have previously been implicated in IBC, and 62% of the miRNA dysregulated in the HN-DCIS comparison are directionally concordant with miRNA dysregulated in IBC. This work identifies a role for these previously implicated miRNA at an early stage of breast cancer development. For example, we found that miR-145 expression was underexpressed in the DCIS-HN comparison. Using an *in situ *hybridization approach, Sempere *et al*. [14] found that miR-145 was restricted to the myoepithelial/basal cell compartment of normal mammary ducts and lobules and was reduced or absent in matching tumor specimens. This finding lends support to our discovery of decreased miR-145 in DCIS, because we know that our epithelial samples include myoepithelial cells.

Although we observed a high concordance rate with previous reports in IBC, in contrast to the seminal study by Iorio *et al*. [13], which examined miRNA expression in bulk tumor tissue versus normal tissue, miR-155 was identified as highly overexpressed in breast tumor tissues. However, miR-155 was not differentially expressed in any of our comparisons. This suggests that either miR-155 is an invasive, specific miRNA or its expression is not epithelium-specific, and also that it was detected because of the heterogeneous cell population presenting bulk tumor tissue. miR-155 has since been described in immune cell function, which supports the latter scenario [51]. Comparison of our data set to others may shed light on other miRNA whose expression is specific to either cancer stage or a particular cell type.

The identification of miRNA targets is crucial to the understanding of their biological role. We hypothesized that there is a coordinate mechanism of dysregulation between the abnormal expression of miRNA and target mRNA in very early breast tumorigenesis. By combining miRNA and gene expression data and integrating miRNA target prediction, we obtained a set of candidate miRNA:mRNA target pairs. Approximately one-half of these target pairs were coordinately expressed and are either false-positive predictions or may in fact positively regulate the target mRNA, albeit a less well-understood phenomenon. However, several instances of miRNA-positive regulation of a target gene have been described [52-54]. In addition, it has been noted that two classes of miRNA network motifs, corresponding to positive and negative regulation of a miRNA and its target, may coexist, and in neuronal cells miRNA tend to be coexpressed in the same direction as their target genes [55]. This may in part explain our observations, although further studies are needed.

Our approach has identified many potentially important early-acting, cancer-promoting mRNA targets, and miRNA dysregulation is a potential mechanism causing these early mRNA changes. Many of the identified target genes have known cancer or anticancer activity. For example, *TXNIP *(thioredoxin-interacting protein), *EGR1, CBX7, HOXA9 *and *FOXN3 *(checkpoint repressor 1) have tumor suppressor functions and are targeted by the potentially oncogenic miRNA miR-93, miR-183, miR-181b, miR-182 and miR-7. Similarly, *WWP1, SDC1 *(syndecan 1), *EZH2, CCND1, ADAM9 *and *MEMO1 *have oncogenic activities and are targeted by the potentially tumor suppressor miRNA miR-195, miR-10b, let-7c, miR-17 and miR-125b.

Many of these target pairs are likely to be relevant to cancer in general and breast cancer in particular; however, we could validate only a subset of these. We found that with modulation of miR-125b, miR-182 and miR-183 expression, we obtained results that suggest these miRNA do regulate the expression of their predicted target genes. The expression of miR-125b is reduced in many cancers, including breast cancer [13,56] and serous ovarian carcinoma [57]. In addition, it has been established that miR-125b targets ErbB2/Her2, and by also targeting *MEMO1*, which interacts with ErbB2/Her2, miR-125b is regulating two functionally related genes. miR-182 and miR-183 are clustered at 7q31.2, a region that is frequently amplified in melanoma [58], and both miRNA are commonly codysregulated in many cancers, including prostate, colon and breast cancer [33,59,60]. In this study, we found that by suppressing the expression of miR-182 and miR-183 *in vitro*, the expression of their four predicted targets, *CBX7, DOK4, NMT2 *and *EGR1*, were upregulated. Two of these, *CBX7 *and *EGR1*, have well-described tumor suppressor functions, and recently *DOK4 *family members (*DOK1, DOK2*, and *DOK3*) were identified as lung tumor suppressors [61]. In addition, the secondary effect of miR-182 repression resulting in upregulation of E-cadherin through CBX7, which we have shown, may have important implications in reversing epithelial neoplasias to a more normal state. Furthermore, in future studies, combined modulation of miR-125b, miR-182 and/or miR-183, as well as other miRNA altered in DCIS, may be effective in reversing the forward progression to IBC.

Admittedly, our study has several limitations, most notably the small sample size and the inclusion of only ER- and PR-positive DCIS. With the use of microdissected, paired breast tissue samples and robust statistical analysis, we sought to minimize potential biases elicited by small the sample size. In fact, the many similarities between our miRNA expression profile of DCIS and others' miRNA expression profiling of IBC suggest that our results are reliable. However, an expansion of this study to include other histological categories could identify subtype-specific dysregulated miRNA.

## Conclusions

The present study provides the first report of a miRNA expression profile in normal breast epithelium and the first integrated analysis of miRNA and mRNA expression in paired samples of histologically normal and preinvasive breast cancer. We have further demonstrated, by modulating the expression of several miRNA, that the expression of their predicted target genes is affected. Taken together, these findings support our hypothesis that changes in miRNA expression in early breast cancer may control many of the parallel changes in gene expression at this stage. This work also implicates the loss of the tumor suppressor miR-125b and the gain of the oncogenic miRNA miR-182 and miR-183 as major contributors to early breast cancer development. Additionally, this study has revealed novel candidate markers of preinvasive breast cancer, which could contribute to the identification of new diagnostic and therapeutic targets. The miRNA and miRNA:mRNA target pairs identified in this study are natural candidates for future investigations.

## Abbreviations

*CBX7*: chromobox homolog 7; DCIS: ductal carcinoma *in situ*; *DOK4*: docking protein 4; *EGR1*: early growth response 1; HN: histologically normal; *MEMO1*: mediator of ErbB2-driven cell motility; miRNA: microRNA; mRNA: messenger RNA; *NMT2*: N-myristoyltransferase 2; *NRIP1/RIP140*: nuclear receptor-interacting protein 1; RM: reduction mammoplasty; RT-qPCR: real-time quantitative polymerase chain reaction; PRM: pooled reduction mammoplasty.

## Competing interests

The authors declare that they have no competing interests.

## Authors' contributions

BNH conceived of and designed the study; executed miRNA expression profiling, subset of gene expression profiling, target prediction and target validation; and drafted the manuscript. PS participated in the design of the study and performed gene and miRNA expression statistical analysis. AdlM reviewed and identified lesions on all histological slides. JL provided expert technical advice and helped to design the validation experiments. CLR conceived of the study, participated in its design and coordination and helped to draft the manuscript. All authors read and approved the final manuscript.

## Supplementary Material

Additional file 1 **LCM series of representative breast epithelial lesions. **Lesions were obtained from healthy normal (RM), histologically normal (HN) and paired adjacent ductal carcinoma *in situ *(DCIS). Lesions were microdissected from 10-μm-thick consecutive tissue sections. *Left-right*: standard hematoxylin and eosin (H&E)-stained "guide slide," diluted H&E-stained precapture and postcapture stromal compartments and captured epithelial compartment. Original magnification, ×40. Click here for file

Additional file 2 ***x-y *scatter correlation plot of triplicate PRM samples. **The cycle threshold (C_T_) values for each of the 385 assays from the three replicate PRM samples are plotted against one another. *R*^2 ^= 0.88, 0.91 and 0.90 (mean = 0.90), respectively, and Pearson's correlation coefficients were 0.94, 0.96 and 0.95 (mean = 0.95), respectively. Click here for file

Additional file 3 List of microRNA considered present or absent in each histological group. Click here for file

Additional file 4 **Expression profiling heatmap of 35 microRNA (miRNA) differentially expressed between PRM, HN and DCIS. **Hierarchical clustering heatmap representation of 35 miRNA overexpressed (red) and underexpressed (green) between PRM, HN and DCIS (*P *< 0.005, fold change >3), sorted by physical (chromosomal) position from top to bottom. Black indicates no change in expression. Brackets indicate the 16 clustered miRNA. *Rows*, miRNA; *columns*, profiled patient samples; *shaded boxes*, lesion type or replicate sample. Click here for file

Additional file 5 All genes significantly differentially expressed in paired HN and DCIS. Click here for file

Additional file 6 Expression correlation of microRNA and coordinately expressed predicted targets. Click here for file

Additional file 7 Enriched gene ontology terms for predicted target genes. Click here for file

Additional file 8 Cancer-specific functional annotation and pathway analysis of predicted target genes. Click here for file

## References

[B1] Horner MJ Ries LAG Krapcho M Neyman N Aminou R Howlader N Altekruse SF Feuer EJ Huang L Mariotto A Miller BA Lewis DR Eisner MP Stinchcomb DG Edwards BK SEER Cancer Statistics Review, 1975-2006 2008 Bethesda, MD: National Cancer Institute

[B2] Guo H Ingolia NT Weissman JS Bartel DP Mammalian microRNAs predominantly act to decrease target mRNA levels Nature 2010 466 835 840 10.1038/nature09267 20703300 PMC2990499

[B3] Krützfeldt J Stoffel M MicroRNAs: a new class of regulatory genes affecting metabolism Cell Metab 2006 4 9 12 16814728 10.1016/j.cmet.2006.05.009

[B4] Nimmo RA Slack FJ An elegant miRror: microRNAs in stem cells, developmental timing and cancer Chromosoma 2009 118 405 418 10.1007/s00412-009-0210-z 19340450 PMC4322900

[B5] Cheng AM Byrom MW Shelton J Ford LP Antisense inhibition of human miRNAs and indications for an involvement of miRNA in cell growth and apoptosis Nucleic Acids Res 2005 33 1290 1297 10.1093/nar/gki200 15741182 PMC552951

[B6] Taft RJ Pang KC Mercer TR Dinger M Mattick JS Non-coding RNAs: regulators of disease J Pathol 2010 220 126 139 10.1002/path.2638 19882673

[B7] Lu J Getz G Miska E Alvarez-Saavedra E Lamb J Peck D Sweet-Cordero A Ebert B Mak R Ferrando A Downing J Jacks T Horvitz H Golub T MicroRNA expression profiles classify human cancers Nature 2005 435 834 838 10.1038/nature03702 15944708

[B8] Friedman RC Farh KK Burge CB Bartel DP Most mammalian mRNAs are conserved targets of microRNAs Genome Res 2009 19 92 105 10.1101/gr.082701.108 18955434 PMC2612969

[B9] Griffiths-Jones S Grocock RJ van Dongen S Bateman A Enright AJ miRBase: microRNA sequences, targets and gene nomenclature Nucleic Acids Res 2006 34 D140 D144 10.1093/nar/gkj112 16381832 PMC1347474

[B10] Lim LP Lau NC Garrett-Engele P Grimson A Schelter JM Castle J Bartel DP Linsley PS Johnson JM Microarray analysis shows that some microRNAs downregulate large numbers of target mRNAs Nature 2005 433 769 773 10.1038/nature03315 15685193

[B11] Mattie MD Benz CC Bowers J Sensinger K Wong L Scott GK Fedele V Ginzinger D Getts R Haqq C Optimized high-throughput microRNA expression profiling provides novel biomarker assessment of clinical prostate and breast cancer biopsies Mol Cancer 2006 5 24 10.1186/1476-4598-5-24 16784538 PMC1563474

[B12] Blenkiron C Goldstein LD Thorne NP Spiteri I Chin SF Dunning MJ Barbosa-Morais NL Teschendorff AE Green AR Ellis IO Tavaré S Caldas C Miska EA MicroRNA expression profiling of human breast cancer identifies new markers of tumor subtype Genome Biol 2007 8 R214 10.1186/gb-2007-8-10-r214 17922911 PMC2246288

[B13] Iorio MV Ferracin M Liu CG Veronese A Spizzo R Sabbioni S Magri E Pedriali M Fabbri M Campiglio M Ménard S Palazzo JP Rosenberg A Musiani P Volinia S Nenci I Calin GA Querzoli P Negrini M Croce CM MicroRNA gene expression deregulation in human breast cancer Cancer Res 2005 65 7065 7070 10.1158/0008-5472.CAN-05-1783 16103053

[B14] Sempere LF Christensen M Silahtaroglu A Bak M Heath CV Schwartz G Wells W Kauppinen S Cole CN Altered MicroRNA expression confined to specific epithelial cell subpopulations in breast cancer Cancer Res 2007 67 11612 11620 10.1158/0008-5472.CAN-07-5019 18089790

[B15] Emery LA Tripathi A King C Kavanah M Mendez J Stone MD de las Morenas A Sebastiani P Rosenberg CL Early dysregulation of cell adhesion and extracellular matrix pathways in breast cancer progression Am J Pathol 2009 175 1292 1302 10.2353/ajpath.2009.090115 19700746 PMC2731147

[B16] Tripathi A King C de la Morenas A Perry VK Burke B Antoine GA Hirsch EF Kavanah M Mendez J Stone M Gerry NP Lenburg ME Rosenberg CL Gene expression abnormalities in histologically normal breast epithelium of breast cancer patients Int J Cancer 2008 122 1557 1566 10.1002/ijc.23267 18058819

[B17] King C Guo N Frampton GM Gerry NP Lenburg ME Rosenberg CL Reliability and reproducibility of gene expression measurements using amplified RNA from laser-microdissected primary breast tissue with oligonucleotide arrays J Mol Diagn 2005 7 57 64 10.1016/S1525-1578(10)60009-8 15681475 PMC1867505

[B18] Li XF Yan PJ Shao ZM Downregulation of miR-193b contributes to enhance urokinase-type plasminogen activator (uPA) expression and tumor progression and invasion in human breast cancer Oncogene 2009 28 3937 3948 10.1038/onc.2009.245 19701247

[B19] Churchill GA Fundamentals of experimental design for cDNA microarrays Nat Genet 2002 32 Suppl 490 495 10.1038/ng1031 12454643

[B20] Gentleman RC Carey VJ Bates DM Bolstad B Dettling M Dudoit S Ellis B Gautier L Ge Y Gentry J Hornik K Hothorn T Huber W Iacus S Irizarry R Leisch F Li C Maechler M Rossini AJ Sawitzki G Smith C Smyth G Tierney L Yang JY Zhang J Bioconductor: open software development for computational biology and bioinformatics Genome Biol 2004 5 R80 10.1186/gb-2004-5-10-r80 15461798 PMC545600

[B21] Graham K de las Morenas A Tripathi A King C Kavanah M Mendez J Stone M Slama J Miller M Antoine G Willers H Sebastiani P Rosenberg CL Gene expression in histologically normal epithelium from breast cancer patients and from cancer-free prophylactic mastectomy patients shares a similar profile Br J Cancer 2010 102 1284 1293 10.1038/sj.bjc.6605576 20197764 PMC2855998

[B22] BADGE (Bayesian Analysis of Differential Gene Expression) http://dcommon.bu.edu/xmlui/handle/2144/1289

[B23] Creighton CJ Nagaraja AK Hanash SM Matzuk MM Gunaratne PH A bioinformatics tool for linking gene expression profiling results with public databases of microRNA target predictions RNA 2008 14 2290 2296 10.1261/rna.1188208 18812437 PMC2578856

[B24] Krek A Grün D Poy M Wolf R Rosenberg L Epstein E MacMenamin P da Piedade I Gunsalus K Stoffel M Rajewsky N Combinatorial microRNA target predictions Nat Genet 2005 37 495 500 10.1038/ng1536 15806104

[B25] Lewis BP Shih IH Jones-Rhoades MW Bartel DP Burge CB Prediction of mammalian microRNA targets Cell 2003 115 787 798 10.1016/S0092-8674(03)01018-3 14697198

[B26] Betel D Wilson M Gabow A Marks DS Sander C The microRNA.org resource: targets and expression Nucleic Acids Res 2008 36 D149 D153 10.1093/nar/gkm995 18158296 PMC2238905

[B27] Huang da W Sherman BT Lempicki RA Systematic and integrative analysis of large gene lists using DAVID bioinformatics resources Nat Protoc 2009 4 44 57 10.1038/nprot.2008.211 19131956

[B28] Baskerville S Bartel DP Microarray profiling of microRNAs reveals frequent coexpression with neighboring miRNAs and host genes RNA 2005 11 241 247 10.1261/rna.7240905 15701730 PMC1370713

[B29] Scott GK Goga A Bhaumik D Berger CE Sullivan CS Benz CC Coordinate suppression of *ERBB2 *and *ERBB3 *by enforced expression of micro-RNA *miR-125a *or *miR-125b* J Biol Chem 2007 282 1479 1486 10.1074/jbc.M609383200 17110380

[B30] Saito Y Liang G Egger G Friedman JM Chuang JC Coetzee GA Jones PA Specific activation of microRNA-127 with downregulation of the proto-oncogene *BCL6 *by chromatin-modifying drugs in human cancer cells Cancer Cell 2006 9 435 443 10.1016/j.ccr.2006.04.020 16766263

[B31] Yan LX Huang XF Shao Q Huang MY Deng L Wu QL Zeng YX Shao JY MicroRNA miR-21 overexpression in human breast cancer is associated with advanced clinical stage, lymph node metastasis and patient poor prognosis RNA 2008 18812439 10.1261/rna.1034808 PMC2578865

[B32] Yu Z Wang C Wang M Li Z Casimiro MC Liu M Wu K Whittle J Ju X Hyslop T McCue P Pestell RG A cyclin D1/microRNA 17/20 regulatory feedback loop in control of breast cancer cell proliferation J Cell Biol 2008 182 509 517 10.1083/jcb.200801079 18695042 PMC2500136

[B33] Guttilla IK White BA Coordinate regulation of FOXO1 by miR-27a, miR-96, and miR-182 in breast cancer cells J Biol Chem 2009 284 23204 23216 10.1074/jbc.M109.031427 19574223 PMC2749094

[B34] Zhang L Huang J Yang N Greshock J Megraw M Giannakakis A Liang S Naylor T Barchetti A Ward M Yao G Medina A O'Brien-Jenkins A Katsaros D Hatzigeorgiou A Gimotty P Weber B Coukos G microRNAs exhibit high frequency genomic alterations in human cancer Proc Natl Acad Sci USA 2006 103 9136 9141 10.1073/pnas.0508889103 16754881 PMC1474008

[B35] Zhang H Su SB Zhou QM Lu YY [Differential expression profiles of microRNAs between breast cancer cells and mammary epithelial cells] [in Chinese] Ai Zheng 2009 28 493 499 19624877

[B36] Frankel LB Christoffersen NR Jacobsen A Lindow M Krogh A Lund AH Programmed cell death 4 (PDCD4) is an important functional target of the microRNA *miR-21 *in breast cancer cells J Biol Chem 2008 283 1026 1033 10.1074/jbc.M707224200 17991735

[B37] Gregory PA Bert AG Paterson EL Barry SC Tsykin A Farshid G Vadas MA Khew-Goodall Y Goodall GJ The miR-200 family and miR-205 regulate epithelial to mesenchymal transition by targeting ZEB1 and SIP1 Nat Cell Biol 2008 10 593 601 10.1038/ncb1722 18376396

[B38] Foekens JA Sieuwerts AM Smid M Look MP de Weerd V Boersma AW Klijn JG Wiemer EA Martens JW Four miRNAs associated with aggressiveness of lymph node-negative, estrogen receptor-positive human breast cancer Proc Natl Acad Sci USA 2008 105 13021 13026 10.1073/pnas.0803304105 18755890 PMC2529088

[B39] Ma L Teruya-Feldstein J Weinberg RA Tumour invasion and metastasis initiated by microRNA-10b in breast cancer Nature 2007 449 682 688 10.1038/nature06174 17898713

[B40] Volinia S Calin GA Liu CG Ambs S Cimmino A Petrocca F Visone R Iorio M Roldo C Ferracin M Prueitt RL Yanaihara N Lanza G Scarpa A Vecchione A Negrini M Harris CC Croce CM A microRNA expression signature of human solid tumors defines cancer gene targets Proc Natl Acad Sci USA 2006 103 2257 2261 10.1073/pnas.0510565103 16461460 PMC1413718

[B41] Si ML Zhu S Wu H Lu Z Wu F Mo YY miR-21-mediated tumor growth: suppression of tumor growth by anti-miR-21 Oncogene 2007 26 2799 2803 10.1038/sj.onc.1210083 17072344

[B42] Fujita S Ito T Mizutani T Minoguchi S Yamamichi N Sakurai K Iba H *miR-21 *gene expression triggered by AP-1 is sustained through a double-negative feedback mechanism J Mol Biol 2008 378 492 504 10.1016/j.jmb.2008.03.015 18384814

[B43] Xu T Zhu Y Xiong Y Ge YY Yun JP Zhuang SM MicroRNA-195 suppresses tumorigenicity and regulates G1/S transition of human hepatocellular carcinoma cells Hepatology 2009 50 113 121 10.1002/hep.22919 19441017

[B44] Qiu C Lienhard S Hynes NE Badache A Leahy DJ Memo is homologous to nonheme iron dioxygenases and binds an ErbB2-derived phosphopeptide in its vestigial active site J Biol Chem 2008 283 2734 2740 10.1074/jbc.M703523200 18045866

[B45] Cavaillès V Dauvois S L'Horset F Lopez G Hoare S Kushner PJ Parker MG Nuclear factor RIP140 modulates transcriptional activation by the estrogen receptor EMBO J 1995 14 3741 3751 7641693 10.1002/j.1460-2075.1995.tb00044.x PMC394449

[B46] Federico A Pallante P Bianco M Ferraro A Esposito F Monti M Cozzolino M Keller S Fedele M Leone V Troncone G Chiariotti L Pucci P Fusco A Chromobox protein homologue 7 protein, with decreased expression in human carcinomas, positively regulates E-cadherin expression by interacting with the histone deacetylase 2 protein Cancer Res 2009 69 7079 7087 10.1158/0008-5472.CAN-09-1542 19706751

[B47] Itoh S Lemay S Osawa M Che W Duan Y Tompkins A Brookes PS Sheu SS Abe J Mitochondrial Dok-4 recruits Src kinase and regulates NF-κB activation in endothelial cells J Biol Chem 2005 280 26383 26396 10.1074/jbc.M410262200 15855164

[B48] Bedirian A Baldwin C Abe J Takano T Lemay S Pleckstrin homology and phosphotyrosine-binding domain-dependent membrane association and tyrosine phosphorylation of Dok-4, an inhibitory adapter molecule expressed in epithelial cells J Biol Chem 2004 279 19335 19349 10.1074/jbc.M310689200 14963042

[B49] Selvakumar P Pasha MK Ashakumary L Dimmock JR Sharma RK Myristoyl-CoA:protein N-myristoyltransferase: a novel molecular approach for cancer therapy Int J Mol Med 2002 10 493 500 12239600

[B50] Baron V Adamson ED Calogero A Ragona G Mercola D The transcription factor Egr1 is a direct regulator of multiple tumor suppressors including TGFβ1, PTEN, p53, and fibronectin Cancer Gene Ther 2006 13 115 124 10.1038/sj.cgt.7700896 16138117 PMC2455793

[B51] Rodriguez A Vigorito E Clare S Warren M Couttet P Soond D van Dongen S Grocock R Das P Miska E Vetrie D Okkenhaug K Enright A Dougan G Turner M Bradley A Requirement of bic/microRNA-155 for normal immune function Science 2007 316 608 611 10.1126/science.1139253 17463290 PMC2610435

[B52] Vasudevan S Tong Y Steitz JA Switching from repression to activation: microRNAs can up-regulate translation Science 2007 318 1931 1934 10.1126/science.1149460 18048652

[B53] Tsai NP Lin YL Wei LN MicroRNA mir-346 targets the 5'-untranslated region of receptor-interacting protein 140 (RIP140) mRNA and up-regulates its protein expression Biochem J 2009 424 411 418 10.1042/BJ20090915 19780716

[B54] Place RF Li LC Pookot D Noonan EJ Dahiya R MicroRNA-373 induces expression of genes with complementary promoter sequences Proc Natl Acad Sci USA 2008 105 1608 1613 10.1073/pnas.0707594105 18227514 PMC2234192

[B55] Tsang J Zhu J van Oudenaarden A MicroRNA-mediated feedback and feedforward loops are recurrent network motifs in mammals Mol Cell 2007 26 753 767 10.1016/j.molcel.2007.05.018 17560377 PMC2072999

[B56] Hui AB Shi W Boutros PC Miller N Pintilie M Fyles T McCready D Wong D Gerster K Waldron L Jurisica I Penn LZ Liu FF Robust global micro-RNA profiling with formalin-fixed paraffin-embedded breast cancer tissues Lab Invest 2009 89 597 606 10.1038/labinvest.2009.12 19290006

[B57] Nam EJ Yoon H Kim SW Kim H Kim YT Kim JH Kim JW Kim S MicroRNA expression profiles in serous ovarian carcinoma Clin Cancer Res 2008 14 2690 2695 10.1158/1078-0432.CCR-07-1731 18451233

[B58] Segura MF Hanniford D Menendez S Reavie L Zou X Alvarez-Diaz S Zakrzewski J Blochin E Rose A Bogunovic D Polsky D Wei J Lee P Belitskaya-Levy I Bhardwaj N Osman I Hernando E Aberrant miR-182 expression promotes melanoma metastasis by repressing FOXO3 and microphthalmia-associated transcription factor Proc Natl Acad Sci USA 2009 106 1814 1819 10.1073/pnas.0808263106 19188590 PMC2634798

[B59] Sarver AL French AJ Borralho PM Thayanithy V Oberg AL Silverstein KA Morlan BW Riska SM Boardman LA Cunningham JM Subramanian S Wang L Smyrk TC Rodrigues CM Thibodeau SN Steer CJ Human colon cancer profiles show differential microRNA expression depending on mismatch repair status and are characteristic of undifferentiated proliferative states BMC Cancer 2009 9 401 10.1186/1471-2407-9-401 19922656 PMC2787532

[B60] Schaefer A Jung M Mollenkopf HJ Wagner I Stephan C Jentzmik F Miller K Lein M Kristiansen G Jung K Diagnostic and prognostic implications of microRNA profiling in prostate carcinoma Int J Cancer 2010 126 1166 1176 19676045 10.1002/ijc.24827

[B61] Berger AH Niki M Morotti A Taylor BS Socci ND Viale A Brennan C Szoke J Motoi N Rothman PB Teruya-Feldstein J Gerald WL Ladanyi M Pandolfi PP Identification of *DOK *genes as lung tumor suppressors Nat Genet 2010 42 216 223 10.1038/ng.527 20139980 PMC2956443

[B62] Zhao Y Deng C Wang J Xiao J Gatalica Z Recker RR Xiao GG Let-7 family miRNAs regulate estrogen receptor alpha signaling in estrogen receptor positive breast cancer Breast Cancer Res Treat 2010 in press 10.1007/s10549-010-0972-2 20535543

