# Supplementary material for: Expression of microRNA and their gene targets are dysregulated in preinvasive breast cancer
Source: Breast Cancer Res. 2011 Mar 4;13(2):R24. doi: 10.1186/bcr2839 (PMC3219184; doi:10.1186/bcr2839)
Supplement: Additional file 1 — LCM series of representative breast epithelial lesions. Lesions were obtained from healthy normal (RM), histologically normal (HN) and paired adjacent ductal carcinoma in situ (DCIS). Lesions were microdissected from 10-μm-thick consecutive tissue sections. Left-right: standard hematoxylin and eosin (H&E)-stained "guide slide," diluted H&E-stained precapture and postcapture stromal compartments and captured epithelial compartment. Original magnification, ×40. [file bcr2839-S1.PDF]

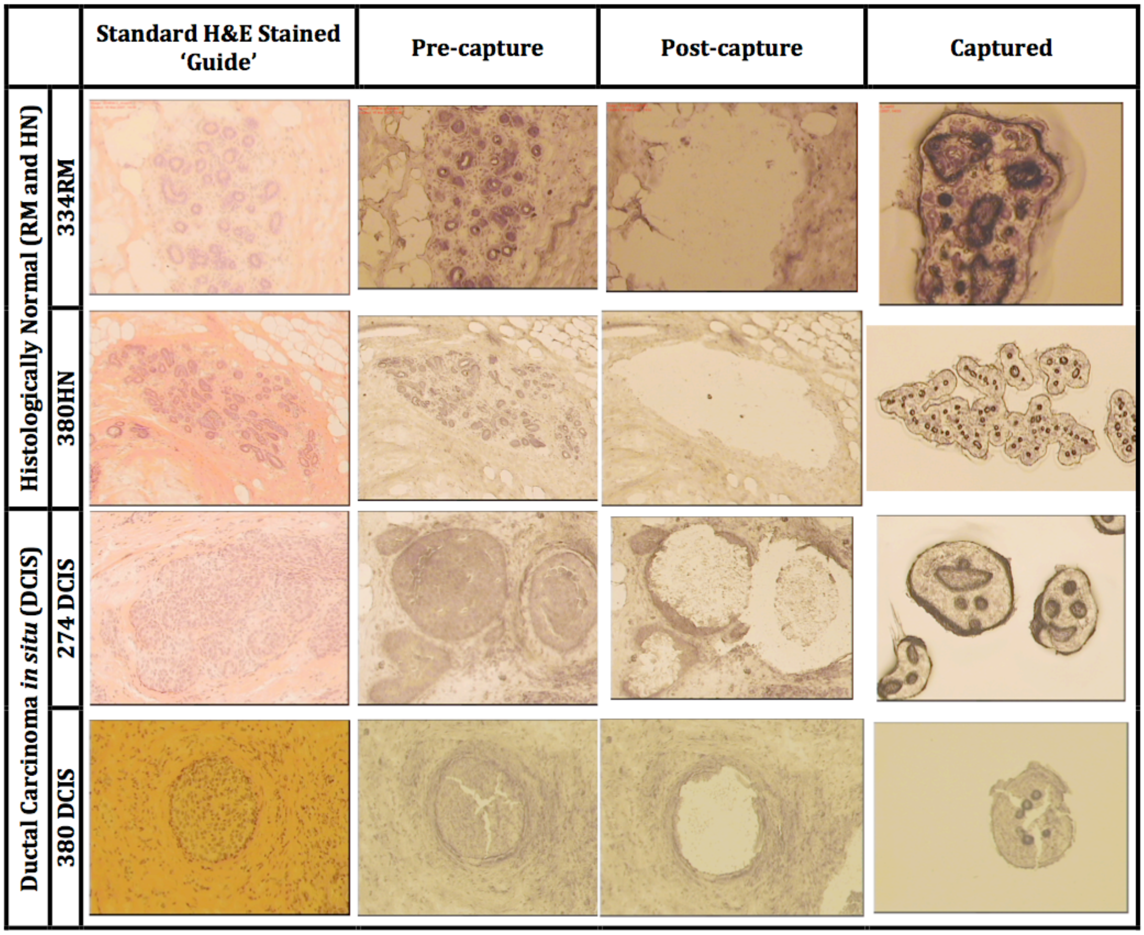

S1. LCM series of representative breast epithelial lesions. Lesions were obtained from healthy normal (RM), histologically normal (HN) and paired adjacent ductal carcinoma *in situ (*DCIS). Lesions were micro-dissected from 10-µm thick consecutive tissue sections; *left-right:* standard hematoxylin and eosin (H&E) stained 'guide slide', dilute H&E stained pre-capture, post-capture stromal compartment, and captured epithelial compartment. 40X magnification.
