# Supplementary material for: Expression of microRNA and their gene targets are dysregulated in preinvasive breast cancer
Source: Breast Cancer Res. 2011 Mar 4;13(2):R24. doi: 10.1186/bcr2839 (PMC3219184; doi:10.1186/bcr2839)
Supplement: Additional file 2 — x-y scatter correlation plot of triplicate PRM samples. The cycle threshold (CT) values for each of the 385 assays from the three replicate PRM samples are plotted against one another. R2 = 0.88, 0.91 and 0.90 (mean = 0.90), respectively, and Pearson's correlation coefficients were 0.94, 0.96 and 0.95 (mean = 0.95), respectively. [file bcr2839-S2.PDF]

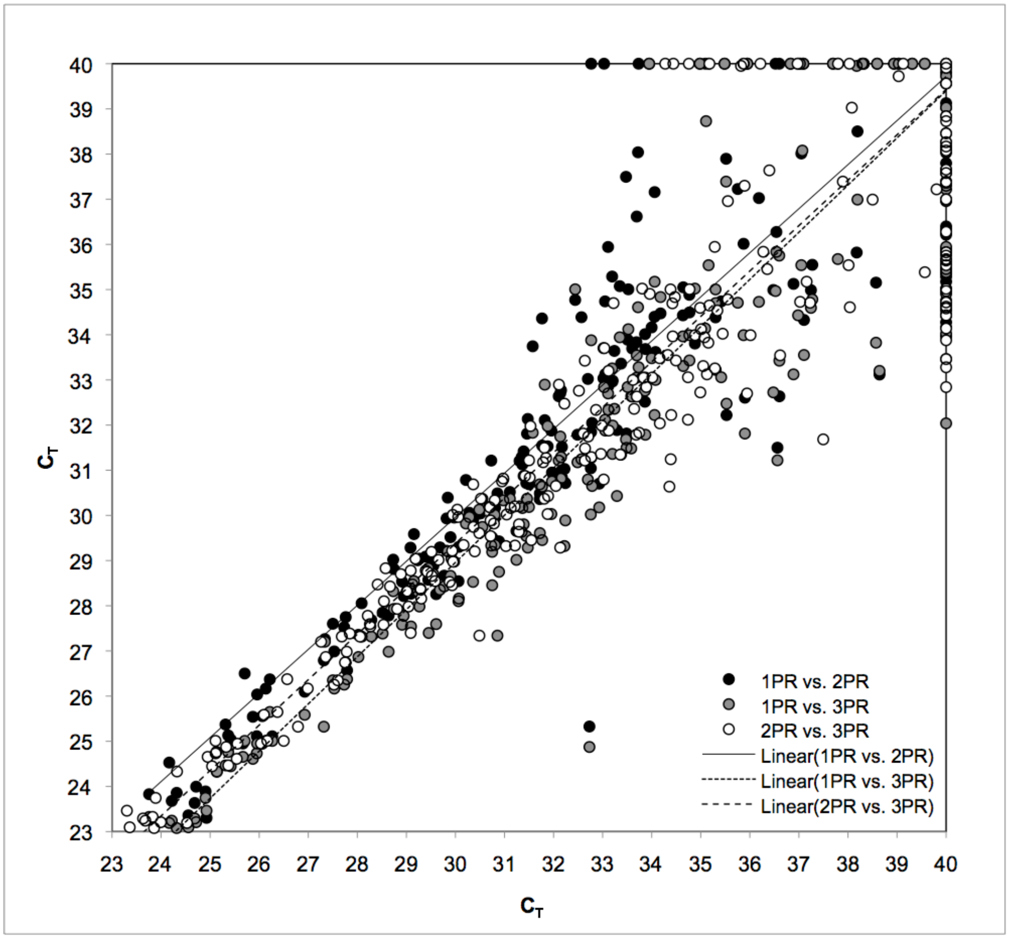

S2 X-Y scatter correlation plot of triplicate PRM samples. To determine the level of correlation between replicate samples, the cycle threshold (C_T) values for each of the 385 assays from each of the 3 replicate PRM samples, are plotted against one another. R^2 =0.88, 0.91, 0.90 (mean=0.90) and Pearson's correlation coefficient of 0.94, 0.96, and 0.95 (mean=0.95)
