# Supplementary material for: Expression of microRNA and their gene targets are dysregulated in preinvasive breast cancer
Source: Breast Cancer Res. 2011 Mar 4;13(2):R24. doi: 10.1186/bcr2839 (PMC3219184; doi:10.1186/bcr2839)
Supplement: Additional file 4 — Expression profiling heatmap of 35 microRNA (miRNA) differentially expressed between PRM, HN and DCIS. Hierarchical clustering heatmap representation of 35 miRNA overexpressed (red) and underexpressed (green) between PRM, HN and DCIS (P < 0.005, fold change >3), sorted by physical (chromosomal) position from top to bottom. Black indicates no change in expression. Brackets indicate the 16 clustered miRNA. Rows, miRNA; columns, profiled patient samples; shaded boxes, lesion type or replicate sample. [file bcr2839-S4.PDF]

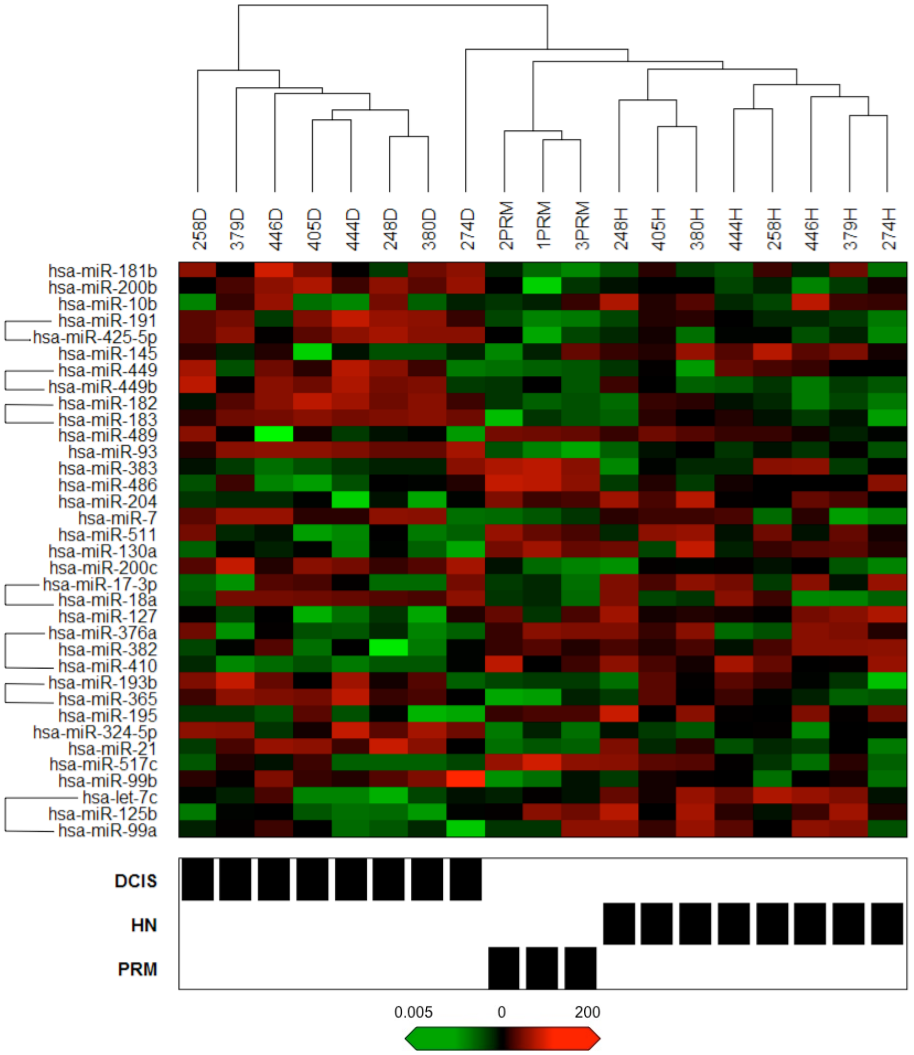

S4. Expression profiling heatmap of 35 miRNAs differentially expressed between PRM, HN and DCIS. Unsupervised hierarchical clustering heat map representation of 35 miRNAs over-expressed (red) and under-expressed (green) between PRM, HN and DCIS ($p<0.005$, fold-change >3), sorted by physical (chromosomal) position from top to bottom, black indicates no change in expression. The 16 clustered miRNAs are indicated by bracketing. *Rows*, miRNAs; *columns*, profiled patient samples; *shaded-boxes*, indicate lesion type or replicate sample.
