# Supplementary material for: Expression of microRNA and their gene targets are dysregulated in preinvasive breast cancer
Source: Breast Cancer Res. 2011 Mar 4;13(2):R24. doi: 10.1186/bcr2839 (PMC3219184; doi:10.1186/bcr2839)
Supplement: Additional file 5 — All genes significantly differentially expressed in paired HN and DCIS. [file bcr2839-S5.PDF]

S5. Table-All genes significantly differentially expressed in paired DCIS *vs.* HN

Affymetrix Probe ID	Mean HN	Mean DCIS	Fold Change	Probability	Gene Name-Gene Description
203108_at	71.10	799.70	9.86	7.13E-05	GPRC5A - G PROTEIN-COUPLED RECEPTOR, FAMILY C, GROUP 5, MEMBER A
220150_s_at	10.99	76.08	6.61	1.57E-03	NEK2 - NIMA (NEVER IN MITOSIS GENE A)-RELATED KINASE 2
208653_s_at	17.11	260.81	5.81	3.68E-04	S100P - S100 CALCIUM BINDING PROTEIN P
211719_x_at	215.63	1628.63	5.14	1.19E-03	FN1 - FIBRONECTIN 1
207828_s_at	188.03	1163.45	5.01	3.13E-03	SCD - STEAROYL-COA DESATURASE (DELTA-9-DESATURASE)
209970_x_at	79.95	408.83	4.73	1.09E-02	RRM2 - RIBONUCLEOTIDE REDUCTASE M2 POLYPEPTIDE
212464_s_at	152.25	1051.54	4.68	1.70E-03	FN1 - FIBRONECTIN 1
205081_at	73.56	532.47	4.51	1.34E-02	CRIP1 - CYSTEINE-RICH PROTEIN 1 (INTESTINAL)
212444_at	151.10	855.14	4.36	1.45E-03	GPRC5A - G PROTEIN-COUPLED RECEPTOR, FAMILY C, GROUP 5, MEMBER A
214858_at	14.57	84.72	4.18	3.03E-03	GPC1 - GLYPICAN 1
216442_x_at	292.94	1464.59	3.94	1.30E-03	FN1 - FIBRONECTIN 1
210495_x_at	297.72	1512.48	3.77	1.01E-03	FN1 - FIBRONECTIN 1
211366_x_at	39.78	177.39	3.53	1.98E-03	RRM2 - RIBONUCLEOTIDE REDUCTASE M2 POLYPEPTIDE
212914_at	26.61	326.20	3.30	9.66E-04	S100A8 - S100 CALCIUM BINDING PROTEIN A8 (CALGRANULIN A)
41660_at	170.36	672.46	3.21	5.03E-03	SCCPDH - SACCHAROPINE DEHYDROGENASE (PUTATIVE)
204508_s_at	170.70	587.92	3.10	4.89E-03	CA12 - CARBONIC ANHYDRASE XII
201818_at	140.90	609.16	3.09	1.04E-03	MUC1 - MUCIN 1, TRANSMEMBRANE
202503_s_at	121.29	421.15	3.05	2.39E-03	KIAA0101 - KIAA0101
201754_at	1155.73	4012.61	3.02	3.90E-03	COX6C - CYTOCHROME C OXIDASE SUBUNIT VIC
221234_s_at	787.75	2490.20	2.93	1.55E-02	MUC1 - MUCIN 1, TRANSMEMBRANE
204567_s_at	42.89	154.79	2.93	8.16E-04	ABCG1 - ATP-BINDING CASSETTE, SUB-FAMILY G (WHITE), MEMBER 1
203963_at	1111.70	3320.33	2.88	3.10E-03	CA12 - CARBONIC ANHYDRASE XII
216442_x_at	155.71	671.00	2.83	4.67E-03	WWP1 - WW DOMAIN CONTAINING E3 UBIQUITIN PROTEIN LIGASE 1
219402_s_at	170.59	501.18	2.81	1.73E-03	DERL1 - DER1-LIKE DOMAIN FAMILY, MEMBER 1
212464_s_at	97.30	309.23	2.79	2.07E-03	WWP1 - WW DOMAIN CONTAINING E3 UBIQUITIN PROTEIN LIGASE 1
214164_x_at	1134.50	3034.68	2.78	3.87E-03	CA12 - CARBONIC ANHYDRASE XII
214290_s_at	100.41	408.95	2.77	4.14E-03	HIST2H2AA3 - HISTONE 2, H2AA3
212150_at	226.59	885.57	2.77	1.25E-02	TFF3 - TREFOIL FACTOR 3 (INTESTINAL)
218546_at	347.42	1226.39	2.75	1.01E-02	RAB31 - RAB31, MEMBER RAS ONCOGENE FAMILY
213001_at	54.18	154.48	2.74	4.03E-03	MELK - MATERNAL EMBRYONIC LEUCINE ZIPPER KINASE
219787_s_at	46.57	160.17	2.69	2.65E-03	ECT2 - EPITHELIAL CELL TRANSFORMING SEQUENCE 2 ONCOGENE
221732_at	44.50	129.98	2.69	2.35E-02	RNF170 - RING FINGER PROTEIN 170
202708_s_at	190.40	581.65	2.66	1.73E-02	HIST1H2BB - HISTONE 1, H2BB
202357_s_at	193.50	723.64	2.65	1.16E-02	CFB - COMPLEMENT FACTOR B
201641_at	254.03	1008.44	2.64	1.47E-02	BST2 - BONE MARROW STROMAL CELL ANTIGEN 2
823_at	182.03	693.45	2.62	1.36E-03	SLC39A6 - SOLUTE CARRIER FAMILY 39 (ZINC TRANSPORTER), MEMBER 6
215867_x_at	1060.96	2602.57	2.53	3.51E-03	CA12 - CARBONIC ANHYDRASE XII
218280_x_at	131.26	436.09	2.53	5.78E-03	HIST1H2BD - HISTONE 1, H2BD
205666_at	390.84	1046.93	2.52	6.75E-03	UGDH - UDP-GLUCOSE DEHYDROGENASE
218992_at	44.44	148.58	2.50	7.89E-03	RBM35A - RNA BINDING MOTIF PROTEIN 35A
203213_at	37.80	97.17	2.45	6.39E-03	CDC2 - CELL DIVISION CYCLE 2, G1 TO S AND G2 TO M
41660_at	124.81	398.87	2.43	2.62E-03	CELSR1 - CADHERIN, EGF LAG SEVEN-PASS G-TYPE RECEPTOR 1 (FLAMINGO HOMOLOG, DROS...
201693_s_at	49.75	126.14	2.41	5.67E-03	THBS1 - THROMBOSPONDIN 1
203881_s_at	184.41	570.77	2.40	1.01E-02	SQLE - SQUALENE EPOXIDASE
208653_s_at	30.97	79.93	2.38	6.68E-03	CD164 - CD164 ANTIGEN, SIALOMUCIN
210735_s_at	47.25	164.36	2.37	1.80E-03	CA12 - CARBONIC ANHYDRASE XII
203358_s_at	42.78	104.86	2.37	5.20E-03	EZH2 - ENHANCER OF ZESTE HOMOLOG 2 (DROSOPHILA)
204170_s_at	104.32	290.42	2.36	4.33E-03	CKS2 - CDC28 PROTEIN KINASE REGULATORY SUBUNIT 2
202723_s_at	199.06	617.09	2.34	2.78E-03	YWHAZ - TYROSINE 3-MONOOXYGENASE/TRYPTOPHAN 5-MONOOXYGENASE ACTIVATION PROTEIN...
218567_x_at	73.86	194.58	2.31	2.26E-02	DPP3 - DIPEPTIDYL-PEPTIDASE 3
212254_s_at	76.81	189.48	2.31	7.04E-03	SYNCRIP - SYNAPTOTAGMIN BINDING, CYTOPLASMIC RNA INTERACTING PROTEIN
213591_at	110.94	291.24	2.30	7.70E-03	NUSAP1 - NUCLEOLAR AND SPINDLE ASSOCIATED PROTEIN 1
208712_at	222.72	529.23	2.30	2.34E-04	CCND1 - CYCLIN D1
200618_at	331.17	939.48	2.29	7.28E-03	LASP1 - LIM AND SH3 PROTEIN 1

219696_at	83.72	315.92	2.28	1.84E-03	RAB31 - RAB31, MEMBER RAS ONCOGENE FAMILY
200621_at	168.20	504.37	2.28	1.14E-03	SLC25A44 - KIAA0446 GENE PRODUCT
209911_x_at	125.07	294.05	2.27	3.34E-03	HIST2H2AA3 - HISTONE 2, H3PS2
200700_s_at	401.39	905.86	2.26	1.04E-02	KDELR2 - KDEL (LYS-ASP-GLU-LEU) ENDOPLASMIC RETICULUM PROTEIN RETENTION RECEPTO...
202956_at	241.57	702.77	2.26	1.94E-03	ARFGEF1 - ADP-RIBOSYLATION FACTOR GUANINE NUCLEOTIDE-EXCHANGE FACTOR 1(BREFELDIN...
210980_s_at	270.66	680.16	2.25	2.05E-02	ASAH1 - N-ACYLSPHINGOSINE AMIDOHYDROLASE (ACID CERAMIDASE) 1
209613_s_at	425.70	1015.56	2.25	1.05E-02	NRIP1 - NUCLEAR RECEPTOR INTERACTING PROTEIN 1
207828_s_at	41.89	116.36	2.25	1.37E-03	CENPF - CENTROMERE PROTEIN F, 350/400KA (MITOSIN)
210201_x_at	232.07	671.35	2.23	9.82E-03	PRSS23 - PROTEASE, SERINE, 23
202543_s_at	113.70	271.87	2.23	6.73E-04	GMFB - GLIA MATURATION FACTOR, BETA
208650_s_at	211.86	509.33	2.22	2.23E-03	CD24 - CD24 ANTIGEN (SMALL CELL LUNG CARCINOMA CLUSTER 4 ANTIGEN)
209047_at	367.06	997.63	2.22	1.03E-02	MICAL2 - MICROTUBULE ASSOCIATED MONOXYGENASE, CALPONIN AND LIM DOMAIN CONTAININ...
214974_x_at	323.65	767.79	2.22	4.06E-03	SLC9A3R1 - SOLUTE CARRIER FAMILY 9 (SODIUM/HYDROGEN EXCHANGER), MEMBER 3 REGULATO...
205066_s_at	160.52	371.61	2.21	1.53E-02	ENPP1 - ECTONUCLEOTIDE PYROPHOSPHATASE/PHOSPHODIESTERASE 1
222155_s_at	37.41	91.75	2.19	1.01E-02	GPR172A - G PROTEIN-COUPLED RECEPTOR 172A
211762_s_at	101.52	264.02	2.18	2.07E-03	KPNA2 - KARYOPHERIN ALPHA 2 (RAG COHORT 1, IMPORTIN ALPHA 1)
220452_x_at	163.12	394.42	2.15	2.17E-03	SCCPDH - SACCHAROPINE DEHYDROGENASE (PUTATIVE)
210652_s_at	299.85	909.11	2.14	1.46E-02	RAB31 - RAB31, MEMBER RAS ONCOGENE FAMILY
201626_at	98.46	235.19	2.13	9.95E-04	INSIG1 - INSULIN INDUCED GENE 1
200776_s_at	91.71	206.76	2.13	6.35E-04	BZW1 - BASIC LEUCINE ZIPPER AND W2 DOMAINS 1
217191_x_at	31.70	80.90	2.13	3.37E-03	COX6CP1 - CYTOCHROME C OXIDASE SUBUNIT VIC PSEUDOGENE 1
203744_at	95.75	250.13	2.13	7.25E-04	HMGB3 - HIGH-MOBILITY GROUP BOX 3
203481_at	276.32	643.70	2.11	1.24E-02	RAB2A - RAB2, MEMBER RAS ONCOGENE FAMILY
208079_s_at	55.79	141.44	2.11	6.38E-03	AURKA - AURORA KINASE A
201818_at	94.41	288.57	2.11	5.43E-03	AYTL2 - ACYLTRANSFERASE LIKE 2
212961_x_at	327.93	746.28	2.10	5.61E-03	SMS - SPERMINE SYNTHASE
210652_s_at	190.92	452.92	2.09	9.34E-03	C1ORF34 - CHROMOSOME 1 OPEN READING FRAME 34
203499_at	46.51	168.37	2.09	4.22E-03	TPD52 - TUMOR PROTEIN D52
203560_at	84.73	242.27	2.08	2.76E-03	GGH - GAMMA-GLUTAMYL HYDROLASE (CONJUGASE, FOLYLPOLYGAMMAGLUTAMYL HYDROLASE)
202655_at	119.10	243.43	2.08	1.43E-02	MOBKL1B - MOB1, MPS ONE BINDER KINASE ACTIVATOR-LIKE 1B (YEAST)
211126_s_at	109.40	277.79	2.08	8.88E-03	SLC31A1 - SOLUTE CARRIER FAMILY 31 (COPPER TRANSPORTERS), MEMBER 1
208091_s_at	260.46	685.63	2.08	6.46E-03	TCEB1 - TRANSCRIPTION ELONGATION FACTOR B (SIII), POLYPEPTIDE 1 (15KDA, ELONGI...
220751_s_at	43.40	109.91	2.08	1.94E-02	RAMP1 - RECEPTOR (CALCITONIN) ACTIVITY MODIFYING PROTEIN 1
202191_s_at	155.16	321.84	2.07	9.95E-03	ZWINT - ZW10 INTERACTOR
204567_s_at	99.29	247.99	2.07	2.48E-03	LPGAT1 - LYSOPHOSPHATIDYLGLYCEROL ACYLTRANSFERASE 1
209283_at	251.07	561.31	2.06	2.06E-03	SLC20A1 - SOLUTE CARRIER FAMILY 20 (PHOSPHATE TRANSPORTER), MEMBER 1
202357_s_at	176.78	421.08	2.06	9.88E-04	SDC1 - SYNDECAN 1
200021_at	2986.48	5753.64	2.05	1.79E-02	CFL1 - COFILIN 1 (NON-MUSCLE)
217191_x_at	116.00	342.50	2.05	4.62E-03	SH3BGRL - SH3 DOMAIN BINDING GLUTAMIC ACID-RICH PROTEIN LIKE
204908_s_at	47.99	99.01	2.05	2.44E-02	BCL3 - B-CELL CLL/LYMPHOMA 3
211712_s_at	63.13	155.88	2.04	9.67E-03	ANXA9 - ANNEXIN A9
202381_at	163.74	361.57	2.04	3.56E-03	ADAM9 - ADAM METALLOPEPTIDASE DOMAIN 9 (MELTRIN GAMMA)
202596_at	928.09	1853.04	2.03	7.18E-03	ENSA - ENDOSULFINE ALPHA
205376_at	121.35	251.73	2.03	1.73E-02	INPP4B - INOSITOL POLYPHOSPHATE-4-PHOSPHATASE, TYPE II, 105KDA
202956_at	440.42	944.90	2.02	6.11E-03	MIRN21 - MICRORNA 21
203042_at	370.90	725.17	2.01	1.85E-02	LAMP2 - LYSOSOMAL-ASSOCIATED MEMBRANE PROTEIN 2
203349_s_at	899.61	2060.20	2.01	1.46E-02	TP63 - TUMOR PROTEIN P73-LIKE
203687_at	3303.73	6482.47	2.00	2.43E-02	SLC39A6 - SOLUTE CARRIER FAMILY 39 (ZINC TRANSPORTER), MEMBER 6
46323_at	293.62	616.98	2.00	3.68E-03	CANT1 - CALCIUM ACTIVATED NUCLEOTIDASE 1
209570_s_at	41.70	87.05	1.99	3.24E-03	SNX24 - SORTING NEXING 24
212738_at	67.33	142.90	1.99	6.20E-03	MLF1IP - MLF1 INTERACTING PROTEIN
202965_s_at	43.60	88.66	1.99	2.30E-02	ROD1 - ROD1 REGULATOR OF DIFFERENTIATION 1 (S. POMBE)

204137_at	116.20	246.70	1.98	8.60E-03	GPR137B - G PROTEIN-COUPLED RECEPTOR 137B
218183_at	146.20	342.84	1.98	5.76E-03	RAB2A - RAB2, MEMBER RAS ONCOGENE FAMILY
204154_at	995.12	2028.41	1.97	7.08E-03	SAT1 - SPERMIDINE/SPERMINE N1-ACETYLTRANSFERASE
221732_at	241.57	548.70	1.97	2.03E-03	CANT1 - CALCIUM ACTIVATED NUCLEOTIDASE 1
218980_at	202.48	394.39	1.96	1.30E-02	UBE2K - HUNTINGTIN INTERACTING PROTEIN 2
217852_s_at	50.74	105.76	1.96	6.50E-03	MOBKL1B - MOB1, MPS ONE BINDER KINASE ACTIVATOR-LIKE 1B (YEAST)
205133_s_at	224.41	493.14	1.96	1.04E-02	HSPE1 - HEAT SHOCK 10KDA PROTEIN 1 (CHAPERONIN 10)
203213_at	139.88	331.91	1.95	1.45E-02	SAR1B - SAR1 GENE HOMOLOG B (S. CEREVISIAE)
218549_s_at	54.71	120.84	1.95	8.43E-03	FAM82B - FAMILY WITH SEQUENCE SIMILARITY 82, MEMBER B
215380_s_at	663.35	1306.26	1.94	1.03E-02	C7ORF24 - CHROMOSOME 7 OPEN READING FRAME 24
37577_at	1482.99	2757.04	1.93	2.36E-02	MLPH - MELANOPHILIN
204017_at	93.28	210.81	1.93	5.92E-03	KDELR3 - KDEL (LYS-ASP-GLU-LEU) ENDOPLASMIC RETICULUM PROTEIN RETENTION RECEPTO...
202934_at	166.11	349.77	1.92	2.04E-03	HK2 - HEXOKINASE 2
211935_at	297.84	620.54	1.92	5.83E-03	ARL6IP1 - ADP-RIBOSYLATION FACTOR-LIKE 6 INTERACTING PROTEIN
204170_s_at	198.19	384.40	1.92	9.93E-03	SEPT2 - SEPTIN 2
217867_x_at	37.64	88.89	1.92	7.76E-03	MUC1 - MUCIN 1, TRANSMEMBRANE
208934_s_at	80.39	168.41	1.91	4.69E-03	LGALS8 - LECTIN, GALACTOSIDE-BINDING, SOLUBLE, 8 (GALECTIN 8)
212706_at	171.09	364.26	1.91	1.06E-02	UGCG - UDP-GLUCOSE CERAMIDE GLUCOSYLTRANSFERASE
215386_at	363.62	830.78	1.91	8.80E-03	POLB - POLYMERASE (DNA DIRECTED), BETA
201437_s_at	157.08	390.13	1.89	1.37E-02	TIAM1 - T-CELL LYMPHOMA INVASION AND METASTASIS 1
204975_at	642.81	1178.56	1.89	2.42E-02	EMP2 - EPITHELIAL MEMBRANE PROTEIN 2
212150_at	256.02	515.83	1.89	1.19E-02	EFR3A - KIAA0143 PROTEIN
209135_at	30.30	63.32	1.89	1.29E-02	MRPL19 - MITOCHONDRIAL RIBOSOMAL PROTEIN L19
214164_x_at	25.28	57.07	1.88	4.53E-03	RIT1 - RAS-LIKE WITHOUT CAAX 1
209135_at	421.74	845.18	1.87	6.42E-03	ASPH - ASPARTATE BETA-HYDROXYLASE
206976_s_at	290.01	527.88	1.87	1.82E-02	HSPH1 - HEAT SHOCK 105KDA/110KDA PROTEIN 1
204359_at	61.48	125.39	1.87	5.65E-03	UGCGL1 - UDP-GLUCOSE CERAMIDE GLUCOSYLTRANSFERASE-LIKE 1
218238_at	136.93	275.97	1.86	3.45E-03	GTPBP4 - GTP BINDING PROTEIN 4
219060_at	95.24	207.81	1.85	1.52E-03	C8ORF32 - CHROMOSOME 8 OPEN READING FRAME 32
213315_x_at	333.75	637.87	1.85	2.19E-04	CXORF40B - CHROMOSOME X OPEN READING FRAME 40A
201437_s_at	118.40	245.60	1.85	9.08E-03	EIF4E - EUKARYOTIC TRANSLATION INITIATION FACTOR 4E
201096_s_at	81.20	160.67	1.85	4.97E-03	ARF4 - ADP-RIBOSYLATION FACTOR 4
211726_s_at	297.99	612.78	1.85	1.02E-02	VEGFA - VASCULAR ENDOTHELIAL GROWTH FACTOR
222212_s_at	381.44	784.50	1.83	4.08E-03	LASS2 - LAG1 LONGEVITY ASSURANCE HOMOLOG 2 (S. CEREVISIAE)
200777_s_at	461.37	852.22	1.83	6.84E-03	PXDN - PEROXIDASIN HOMOLOG (DROSOPHILA)
201341_at	141.40	265.35	1.82	5.60E-03	ENC1 - ECTODERMAL-NEURAL CORTEX (WITH BTB-LIKE DOMAIN)
37577_at	244.11	473.59	1.82	4.33E-03	PGK1 - PHOSPHOGLYCERATE KINASE 1
200853_at	238.10	456.15	1.82	2.37E-02	H2AFZ - H2A HISTONE FAMILY, MEMBER Z
201091_s_at	76.59	151.38	1.81	1.33E-03	CBX3 - CHROMOBOX HOMOLOG 3 (HP1 GAMMA HOMOLOG, DROSOPHILA)
222352_at	46.06	87.21	1.81	4.95E-03	TSPAN3 - TETRASPANIN 3
219787_s_at	407.51	749.70	1.80	9.16E-03	TEGT - TESTIS ENHANCED GENE TRANSCRIPT (BAX INHIBITOR 1)
207030_s_at	265.38	516.98	1.80	1.21E-02	SLC2A10 - SOLUTE CARRIER FAMILY 2 (FACILITATED GLUCOSE TRANSPORTER), MEMBER 10
201091_s_at	241.52	518.84	1.80	1.30E-02	S100A14 - S100 CALCIUM BINDING PROTEIN A14
215438_x_at	343.32	611.30	1.80	8.55E-03	GSPT1 - G1 TO S PHASE TRANSITION 1
213492_at	1008.37	1917.54	1.80	5.38E-03	SERP1 - STRESS-ASSOCIATED ENDOPLASMIC RETICULUM PROTEIN 1
208091_s_at	210.75	394.15	1.79	6.98E-03	ECOP - EGFR-COAMPLIFIED AND OVEREXPRESSED PROTEIN
221802_s_at	377.46	680.32	1.79	1.93E-02	KIAA1598 - KIAA1598
202641_at	912.59	1633.81	1.79	1.35E-02	PGK1 - PHOSPHOGLYCERATE KINASE 1
221935_s_at	223.21	448.27	1.79	2.84E-03	RAD23B - RAD23 HOMOLOG B (S. CEREVISIAE)
212446_s_at	173.31	322.03	1.78	2.08E-02	LASS6 - LAG1 LONGEVITY ASSURANCE HOMOLOG 6 (S. CEREVISIAE)
213800_at	525.80	973.32	1.78	1.76E-02	SDHC - SUCCINATE DEHYDROGENASE COMPLEX, SUBUNIT C, INTEGRAL MEMBRANE PROTEIN,...
205249_at	51.49	100.78	1.78	3.26E-04	THBS1 - THROMBOSPONDIN 1
209770_at	87.16	172.32	1.78	5.20E-03	PTP4A1 - PROTEIN TYROSINE PHOSPHATASE TYPE IVA, MEMBER 1
200777_s_at	154.00	324.15	1.78	6.10E-03	NA
203358_s_at	748.11	1497.03	1.77	5.14E-03	TRIB1 - TRIBBLES HOMOLOG 1 (DROSOPHILA)
218992_at	88.35	168.91	1.77	2.30E-02	C9ORF46 - CHROMOSOME 9 OPEN READING FRAME 46

222041_at	155.32	306.69	1.77	1.32E-02	STK39 - SERINE THREONINE KINASE 39 (STE20/SPS1 HOMOLOG, YEAST)
221896_s_at	199.15	382.99	1.77	1.30E-02	HIGD1A - HIG1 DOMAIN FAMILY, MEMBER 1A
201277_s_at	544.84	925.04	1.76	5.33E-04	HNRPAB - HETEROGENEOUS NUCLEAR RIBONUCLEOPROTEIN A/B
209569_x_at	671.22	1167.75	1.75	1.06E-03	SNRPG - SMALL NUCLEAR RIBONUCLEOPROTEIN POLYPEPTIDE G
201422_at	237.08	479.86	1.75	1.31E-02	IFI30 - INTERFERON, GAMMA-INDUCIBLE PROTEIN 30
204851_s_at	722.97	1383.53	1.75	2.39E-02	SPARC - SECRETED PROTEIN, ACIDIC, CYSTEINE-RICH (OSTEONECTIN)
204437_s_at	241.58	431.11	1.75	5.04E-03	YME1L1 - YME1-LIKE 1 (S. CEREVISIAE)
49452_at	358.34	673.15	1.75	1.32E-02	LSM4 - LSM4 HOMOLOG, U6 SMALL NUCLEAR RNA ASSOCIATED (S. CEREVISIAE)
203575_at	105.78	208.86	1.75	1.86E-03	SLC25A44 - KIAA0446 GENE PRODUCT
215016_x_at	787.24	1304.08	1.74	6.52E-03	TACSTD1 - TUMOR-ASSOCIATED CALCIUM SIGNAL TRANSDUCER 1
210495_x_at	160.54	305.23	1.74	3.14E-03	VPS13B - COHEN SYNDROME 1
200881_s_at	301.86	535.51	1.74	1.11E-02	DNAJA1 - DNAJ (HSP40) HOMOLOG, SUBFAMILY A, MEMBER 1
215867_x_at	65.95	122.49	1.74	4.11E-03	RMI1 - CHROMOSOME 9 OPEN READING FRAME 76
201888_s_at	54.63	99.72	1.73	2.11E-03	IL13RA1 - INTERLEUKIN 13 RECEPTOR, ALPHA 1
213004_at	219.90	398.12	1.73	1.53E-03	MEMO1 - CHROMOSOME 2 OPEN READING FRAME 4
211653_x_at	256.80	464.34	1.73	9.29E-04	MAP7 - MICROTUBULE-ASSOCIATED PROTEIN 7
203963_at	298.18	545.99	1.72	1.57E-02	NME1 - NON-METASTATIC CELLS 1, PROTEIN (NM23A) EXPRESSED IN
204971_at	215.88	381.05	1.71	2.45E-02	SLC35A2 - SOLUTE CARRIER FAMILY 35 (UDP-GALACTOSE TRANSPORTER), MEMBER A2
60474_at	271.58	514.65	1.71	6.03E-03	RAB8A - RAB8A, MEMBER RAS ONCOGENE FAMILY
212961_x_at	344.81	583.41	1.70	1.01E-03	CXORF40B - CHROMOSOME X OPEN READING FRAME 40A
221234_s_at	1167.20	1962.42	1.70	1.42E-03	PRDX1 - PEROXIREDOXIN 1
208687_x_at	794.50	1397.87	1.70	3.62E-03	HSPA8 - HEAT SHOCK 70KDA PROTEIN 8
210347_s_at	74.18	135.30	1.70	7.82E-04	PRKAR1A - PROTEIN KINASE, CAMP-DEPENDENT, REGULATORY, TYPE I, ALPHA (TISSUE SPEC...
209691_s_at	71.42	129.77	1.70	1.56E-02	SSR1 - SIGNAL SEQUENCE RECEPTOR, ALPHA (TRANSLOCON-ASSOCIATED PROTEIN ALPHA)
222108_at	168.11	311.60	1.69	7.17E-03	MB - MYOGLOBIN
219696_at	151.95	277.40	1.69	4.64E-03	C1ORF218 - HYPOTHETICAL PROTEIN FLJ20054
213005_s_at	118.52	200.78	1.69	1.74E-02	PCK2 - PHOSPHOENOLPYRUVATE CARBOXYKINASE 2 (MITOCHONDRIAL)
211931_s_at	857.90	1489.77	1.68	1.57E-04	HNRPA3 - HETEROGENEOUS NUCLEAR RIBONUCLEOPROTEIN A3
202641_at	322.14	551.87	1.68	1.10E-02	ARL3 - ADP-RIBOSYLATION FACTOR-LIKE 3
201096_s_at	150.20	274.39	1.68	1.52E-02	PDIA4 - PROTEIN DISULFIDE ISOMERASE FAMILY A, MEMBER 4
217867_x_at	546.97	969.58	1.67	4.04E-03	BACE2 - BETA-SITE APP-CLEAVING ENZYME 2
201413_at	504.41	884.73	1.67	1.30E-02	HSD17B4 - HYDROXYSTEROID (17-BETA) DEHYDROGENASE 4
203755_at	52.99	90.71	1.66	1.86E-02	BUB1B - BUB1 BUDDING UNINHIBITED BY BENZIMIDAZOLES 1 HOMOLOG BETA (YEAST)
213591_at	569.25	928.27	1.65	1.17E-02	MARCKSL1 - MARCKS-LIKE 1
214112_s_at	285.95	470.77	1.64	1.98E-03	CXCL5 - CHEMOKINE (C-X-C MOTIF) LIGAND 5
202655_at	147.10	248.23	1.64	7.02E-03	ARMET - ARGININE-RICH, MUTATED IN EARLY STAGE TUMORS
205022_s_at	210.72	344.35	1.63	9.98E-03	YME1L1 - YME1-LIKE 1 (S. CEREVISIAE)
200777_s_at	661.09	1078.01	1.62	6.88E-03	BZW1 - BASIC LEUCINE ZIPPER AND W2 DOMAINS 1
201341_at	126.03	212.36	1.62	3.13E-03	TMEM33 - TRANSMEMBRANE PROTEIN 33
221932_s_at	328.99	540.19	1.62	1.05E-02	GLRX5 - GLUTAREDOXIN 5 HOMOLOG (S. CEREVISIAE)
217852_s_at	863.05	1437.16	1.60	5.12E-03	ARL8B - ADP-RIBOSYLATION FACTOR-LIKE 8B
201098_at	219.18	353.80	1.58	1.85E-03	COPB2 - COATOMER PROTEIN COMPLEX, SUBUNIT BETA 2 (BETA PRIME)
203481_at	38.16	66.32	1.58	3.65E-03	C10ORF6 - CHROMOSOME 10 OPEN READING FRAME 6
200881_s_at	363.94	561.44	1.58	3.22E-04	SQRDL - SULFIDE QUINONE REDUCTASE-LIKE (YEAST)
200776_s_at	455.90	741.97	1.57	2.53E-03	RAB11A - RAB11A, MEMBER RAS ONCOGENE FAMILY
213524_s_at	403.55	629.77	1.53	6.66E-04	ZNF706 - ZINC FINGER PROTEIN 706
213540_at	229.24	132.11	0.62	1.30E-02	HSD17B8 - HYDROXYSTEROID (17-BETA) DEHYDROGENASE 8
201117_s_at	89.06	55.18	0.62	4.00E-03	SIPA1L1 - SIGNAL-INDUCED PROLIFERATION-ASSOCIATED 1 LIKE 1
203755_at	186.57	115.19	0.61	2.00E-03	NA
200974_at	787.86	464.45	0.61	4.00E-03	LTBP2 - LATENT TRANSFORMING GROWTH FACTOR BETA BINDING PROTEIN 2
219060_at	76.39	44.10	0.60	3.00E-03	RASL12 - RAS-LIKE, FAMILY 12
211366_x_at	145.73	84.63	0.60	7.00E-03	CASP1 - CASPASE 1, APOPTOSIS-RELATED CYSTEINE PEPTIDASE (INTERLEUKIN 1, BETA, ...
65718_at	95.29	59.40	0.60	1.30E-02	GPR124 - G PROTEIN-COUPLED RECEPTOR 124

219498_s_at	82.28	48.91	0.60	9.00E-03	PRKD3 - PROTEIN KINASE D3
204920_at	78.01	46.94	0.60	3.00E-03	SIRPA - PROTEIN TYROSINE PHOSPHATASE, NON-RECEPTOR TYPE SUBSTRATE 1
218183_at	282.56	162.02	0.59	0.00E+00	NA
215380_s_at	93.37	55.15	0.59	1.90E-02	RARB - RETINOIC ACID RECEPTOR, BETA
204781_s_at	102.07	58.96	0.59	2.00E-03	FAS - FAS (TNF RECEPTOR SUPERFAMILY, MEMBER 6)
204975_at	133.80	74.73	0.59	1.30E-02	TMCO6 - HYPOTHETICAL PROTEIN PRO1580
215513_at	109.44	64.95	0.58	1.10E-02	HYMAI - HYDATIDIFORM MOLE ASSOCIATED AND IMPRINTED
207016_s_at	79.70	50.41	0.58	2.30E-02	NTRK3 - NEUROTROPHIC TYROSINE KINASE, RECEPTOR, TYPE 3
212738_at	68.97	41.60	0.58	1.80E-02	PGAP1 - GPI DEACYLASE
204567_s_at	110.49	63.35	0.58	1.00E-03	NMT2 - N-MYRISTOYLTRANSFERASE 2
204457_s_at	96.92	51.38	0.58	5.00E-03	ZSCAN12 - ZINC FINGER PROTEIN 96
202022_at	97.59	54.18	0.58	1.40E-02	ALDOC - ALDOLASE C, FRUCTOSE-BISPHOSPHATE
219696_at	469.89	255.26	0.58	2.00E-03	NAB1 - NGFI-A BINDING PROTEIN 1 (EGR1 BINDING PROTEIN 1)
200974_at	97.40	57.64	0.58	6.00E-03	NR3C1 - NUCLEAR RECEPTOR SUBFAMILY 3, GROUP C, MEMBER 1 (GLUCOCORTICOID RECEPT...
210201_x_at	160.77	94.16	0.57	7.00E-03	BIN1 - BRIDGING INTEGRATOR 1
215016_x_at	833.30	471.44	0.57	2.00E-02	DST - DYSTONIN
213900_at	113.54	61.06	0.57	1.30E-02	NLRP1 - NACHT, LEUCINE RICH REPEAT AND PYD (PYRIN DOMAIN) CONTAINING 1
204154_at	67.53	36.15	0.57	1.30E-02	CDO1 - CYSTEINE DIOXYGENASE, TYPE I
221447_s_at	140.18	78.79	0.57	7.00E-03	GLT8D2 - GLYCOSYLTRANSFERASE 8 DOMAIN CONTAINING 2
211126_s_at	59.70	33.35	0.57	7.00E-03	CSRP2 - CYSTEINE AND GLYCINE-RICH PROTEIN 2
208079_s_at	146.81	79.61	0.56	4.00E-03	PLAGL1 - PLEIOMORPHIC ADENOMA GENE-LIKE 1
209892_at	71.60	37.54	0.56	1.60E-02	FUT4 - FUCOSYLTRANSFERASE 4 (ALPHA (1,3) FUCOSYLTRANSFERASE, MYELOID-SPECIFIC...
222352_at	57.44	31.29	0.56	5.00E-03	FBXO31 - F-BOX PROTEIN 31
218486_at	185.87	104.45	0.56	7.00E-03	KLF11 - KRUPPEL-LIKE FACTOR 11
204920_at	91.40	52.47	0.56	1.20E-02	CPS1 - CARBAMOYL-PHOSPHATE SYNTHETASE 1, MITOCHONDRIAL
205348_s_at	115.93	54.60	0.56	2.20E-02	DYNC1I1 - DYNEIN, CYTOPLASMIC 1, INTERMEDIATE CHAIN 1
202724_s_at	84.94	45.47	0.56	1.00E-03	ZFHX4 - ZINC FINGER HOMEODOMAIN 4
201641_at	80.64	45.66	0.56	4.00E-03	NA - ,CONSENSUS INCLUDES GB:AV720803 /FEA=EST /DB_XREF=GI:10817955 /DB_XREF
49452_at	670.10	353.64	0.56	1.90E-02	ACACB - ACETYL-COENZYME A CARBOXYLASE BETA
209691_s_at	80.47	43.39	0.56	4.00E-03	DOK4 - DOCKING PROTEIN 4
210105_s_at	183.54	96.89	0.56	1.00E-03	FYN - FYN ONCOGENE RELATED TO SRC, FGR, YES
209892_at	128.08	70.08	0.56	5.00E-03	ZNF204 - ZINC FINGER PROTEIN 204
210105_s_at	159.05	87.94	0.55	1.00E-03	ZNF248 - ZINC FINGER PROTEIN 248
213645_at	551.06	301.54	0.55	2.40E-02	ENOSF1 - ENOLASE SUPERFAMILY MEMBER 1
209970_x_at	198.84	103.79	0.55	1.00E-02	CASP1 - CASPASE 1, APOPTOSIS-RELATED CYSTEINE PEPTIDASE (INTERLEUKIN 1, BETA, ...
204908_s_at	796.47	419.80	0.55	3.00E-03	PRNP - PRION PROTEIN (P27-30) (CREUTZFELD-JAKOB DISEASE, GERSTMANN-STRAUSLER-...
213005_s_at	394.00	208.32	0.55	1.70E-02	ANKRD15 - ANKYRIN REPEAT DOMAIN 15
202972_s_at	112.03	60.41	0.55	1.80E-02	FAM13A1 - FAMILY WITH SEQUENCE SIMILARITY 13, MEMBER A1
212677_s_at	292.16	158.75	0.55	5.00E-03	CEP68 - CENTROSOMAL PROTEIN 68KDA
203065_s_at	204.06	102.71	0.55	2.20E-02	CAV1 - CAVEOLIN 1, CAVEOLAE PROTEIN, 22KDA
216594_x_at	111.43	63.25	0.55	6.00E-03	MAP2K6 - MITOGEN-ACTIVATED PROTEIN KINASE KINASE 6
203499_at	97.86	51.65	0.55	8.00E-03	EPHA2 - EPH RECEPTOR A2
219497_s_at	121.84	61.38	0.54	0.00E+00	PRKD1 - PROTEIN KINASE D1
203969_at	74.27	37.39	0.54	3.00E-03	LOC153914 - HYPOTHETICAL PROTEIN LOC153914
220452_x_at	350.37	179.59	0.54	1.00E-03	CECR7 - CAT EYE SYNDROME CHROMOSOME REGION, CANDIDATE 7
60474_at	57.43	29.53	0.54	1.30E-02	NAP1L3 - NUCLEOSOME ASSEMBLY PROTEIN 1-LIKE 3
217867_x_at	77.37	37.50	0.54	7.00E-03	POU6F1 - POU DOMAIN, CLASS 6, TRANSCRIPTION FACTOR 1
212813_at	324.95	169.62	0.54	0.00E+00	JAM3 - JUNCTIONAL ADHESION MOLECULE 3
211653_x_at	83.30	40.30	0.54	6.00E-03	AKR1C2 - ALDO-KETO REDUCTASE FAMILY 1, MEMBER C2 (DIHYDRODIOL DEHYDROGENASE 2; ...
209770_at	208.62	107.05	0.54	0.00E+00	BTN3A1 - BUTYROPHILIN, SUBFAMILY 3, MEMBER A1
219935_at	108.46	55.76	0.54	2.00E-03	NRG1 - NEUREGULIN 1
219313_at	119.13	64.37	0.54	3.00E-03	GRAMD1C - GRAM DOMAIN CONTAINING 1C

218546_at	352.36	177.11	0.53	1.10E-02	C1ORF115 - CHROMOSOME 1 OPEN READING FRAME 115
205003_at	148.22	80.67	0.53	2.00E-02	DOCK4 - DEDICATOR OF CYTOKINESIS 4
214844_s_at	157.53	92.32	0.53	2.40E-02	DOK5 - DOCKING PROTEIN 5
216594_x_at	158.84	70.41	0.53	3.00E-03	AKR1C1 - ALDO-KETO REDUCTASE FAMILY 1, MEMBER C1 (DIHYDRODIOL DEHYDROGENASE 1; ...
214439_x_at	96.13	51.74	0.53	1.00E-02	BIN1 - BRIDGING INTEGRATOR 1
203963_at	507.03	276.63	0.53	5.00E-03	RGL1 - RAL GUANINE NUCLEOTIDE DISSOCIATION STIMULATOR-LIKE 1
214247_s_at	1347.86	645.19	0.53	7.00E-03	DKK3 - DICKKOPF HOMOLOG 3 (XENOPUS LAEVIS)
222041_at	229.51	115.35	0.53	5.00E-03	DPH1 - DPH1 HOMOLOG (S. CEREVISIAE)
205527_s_at	153.71	68.73	0.52	1.50E-02	GEMIN4 - DKFZP434B131 PROTEIN
211935_at	582.03	273.54	0.52	1.30E-02	PHF17 - PHD FINGER PROTEIN 17
210735_s_at	132.30	72.08	0.52	7.00E-03	RIMS3 - REGULATING SYNAPTIC MEMBRANE EXOCYTOSIS 3
64900_at	66.66	36.00	0.52	1.20E-02	ABHD14B - ABHYDROLASE DOMAIN CONTAINING 14B
210652_s_at	81.91	41.76	0.52	8.00E-03	NAP1L2 - NUCLEOSOME ASSEMBLY PROTEIN 1-LIKE 2
215306_at	82.30	40.15	0.52	2.00E-03	LHCGR - LUTEINIZING HORMONE/CHORIOGONADOTROPIN RECEPTOR
219060_at	2017.28	1093.88	0.52	1.90E-02	NFIB - NUCLEAR FACTOR I/B
215386_at	83.71	46.43	0.52	1.90E-02	AUTS2 - AUTISM SUSCEPTIBILITY CANDIDATE 2
200776_s_at	111.46	54.87	0.52	1.00E-03	NA
213004_at	78.75	44.04	0.51	5.00E-03	ANGPTL2 - ANGIOPOIETIN-LIKE 2
213492_at	110.32	53.92	0.51	7.00E-03	COL2A1 - COLLAGEN, TYPE II, ALPHA 1 (PRIMARY OSTEOARTHRITIS, SPONDYLOEPIPHYSEAL...
219213_at	117.22	58.70	0.51	1.00E-03	JAM2 - JUNCTIONAL ADHESION MOLECULE 2
221935_s_at	86.18	43.25	0.51	1.20E-02	C3ORF64 - HYPOTHETICAL PROTEIN FLJ13078
209770_at	72.26	36.89	0.51	6.00E-03	NA
202655_at	146.06	69.17	0.51	2.00E-02	PIP5K1B - PHOSPHATIDYLINOSITOL-4-PHOSPHATE 5-KINASE, TYPE I, BETA
203323_at	235.76	115.72	0.51	5.00E-03	CAV2 - CAVEOLIN 2
212254_s_at	706.23	327.50	0.51	2.10E-02	DST - DYSTONIN
205022_s_at	195.33	97.14	0.51	0.00E+00	FOXN3 - CHECKPOINT SUPPRESSOR 1
221530_s_at	273.42	124.13	0.50	1.10E-02	PROS1 - PROTEIN S (ALPHA)
203575_at	150.38	71.89	0.50	2.50E-02	CSNK2A2 - CASEIN KINASE 2, ALPHA PRIME POLYPEPTIDE
209883_at	83.92	43.24	0.50	1.50E-02	GLT25D2 - GLYCOSYLTRANSFERASE 25 DOMAIN CONTAINING 2
46323_at	178.86	87.92	0.50	5.00E-03	RNF39 - RING FINGER PROTEIN 39
222108_at	95.82	45.58	0.50	7.00E-03	OLFML1 - OLFACTOMEDIN-LIKE 1
220150_s_at	91.82	46.08	0.50	2.00E-03	C6ORF60 - CHROMOSOME 6 OPEN READING FRAME 60
217852_s_at	1855.45	878.51	0.50	1.40E-02	PIK3R1 - PHOSPHOINOSITIDE-3-KINASE, REGULATORY SUBUNIT 1 (P85 ALPHA)
221530_s_at	463.64	224.51	0.50	2.00E-03	
213001_at	201.09	95.06	0.50	3.00E-03	ANGPTL2 - ANGIOPOIETIN-LIKE 2
205117_at	102.32	44.15	0.49	2.00E-03	TXNIP - THIOREDOXIN INTERACTING PROTEIN
218546_at	162.98	81.78	0.49	4.00E-03	NA
219402_s_at	254.49	95.23	0.49	5.00E-03	SPON1 - SPONDIN 1, EXTRACELLULAR MATRIX PROTEIN
220765_s_at	140.21	64.97	0.49	1.90E-02	LIMS2 - LIM AND SENESCENT CELL ANTIGEN-LIKE DOMAINS 2
218183_at	60.61	27.15	0.49	1.90E-02	C16ORF5 - CHROMOSOME 16 OPEN READING FRAME 5
201117_s_at	216.93	98.65	0.48	5.00E-03	CPE - CARBOXYPEPTIDASE E
222108_at	1369.50	579.48	0.48	9.00E-03	AMIGO2 - ADHESION MOLECULE WITH IG-LIKE DOMAIN 2
215386_at	614.10	246.27	0.48	9.00E-03	MT1F - METALLOTHIONEIN 1F (FUNCTIONAL)
203903_s_at	173.93	81.77	0.48	2.00E-03	HEPH - HEPHAESTIN
202381_at	69.28	34.82	0.48	6.00E-03	LZTS1 - LEUCINE ZIPPER, PUTATIVE TUMOR SUPPRESSOR 1
212706_at	82.42	41.38	0.48	6.00E-03	FLJ21767 - HYPOTHETICAL PROTEIN FLJ21767
214112_s_at	60.93	33.79	0.48	1.40E-02	SNCA - SYNUCLEIN, ALPHA (NON A4 COMPONENT OF AMYLOID PRECURSOR)
221234_s_at	97.52	44.42	0.48	1.00E-02	BACH2 - BTB AND CNC HOMOLOGY 1, BASIC LEUCINE ZIPPER TRANSCRIPTION FACTOR 2
220262_s_at	74.89	38.34	0.47	2.50E-02	DLK2 - EGF-LIKE-DOMAIN, MULTIPLE 9
209569_x_at	102.19	48.10	0.47	0.00E+00	D4S234E - DNA SEGMENT ON CHROMOSOME 4 (UNIQUE) 234 EXPRESSED SEQUENCE
221530_s_at	874.31	409.54	0.47	2.00E-03	BHLHB3 - BASIC HELIX-LOOP-HELIX DOMAIN CONTAINING, CLASS B, 3
210201_x_at	92.06	42.68	0.47	2.00E-03	NA

218656_s_at	414.50	182.87	0.47	1.40E-02	LHFP - LIPOMA HMGIC FUSION PARTNER
202274_at	399.46	188.66	0.47	8.00E-03	NR3C1 - NUCLEAR RECEPTOR SUBFAMILY 3, GROUP C, MEMBER 1 (GLUCOCORTICOID RECEPT...
202022_at	300.52	131.34	0.46	8.00E-03	NXN - NUCLEOREDOXIN
220150_s_at	57.87	29.23	0.46	1.50E-02	RAPGEF2 - RAP GUANINE NUCLEOTIDE EXCHANGE FACTOR (GEF) 2
202724_s_at	299.98	128.15	0.46	0.00E+00	FOXO1 - FORKHEAD BOX O1A (RHABDOMYOSARCOMA)
205498_at	258.20	107.82	0.46	3.00E-03	GHR - GROWTH HORMONE RECEPTOR
220751_s_at	119.58	60.70	0.45	1.00E-02	C5ORF4 - CHROMOSOME 5 OPEN READING FRAME 4
214439_x_at	340.58	146.55	0.45	1.00E-03	NA
204773_at	110.09	50.68	0.45	5.00E-03	IL11RA - INTERLEUKIN 11 RECEPTOR, ALPHA
201540_at	187.88	71.89	0.45	1.00E-02	TYMS - THYMIDYLATE SYNTHETASE
201984_s_at	245.29	100.38	0.45	2.00E-03	TGFB1I1 - TRANSFORMING GROWTH FACTOR BETA 1 INDUCED TRANSCRIPT 1
212914_at	544.86	231.53	0.45	1.50E-02	CBX7 - CHROMOBOX HOMOLOG 7
213524_s_at	79.90	36.79	0.45	5.00E-03	G0S2 - G0/G1SWITCH 2
202966_at	86.58	35.63	0.45	8.00E-03	RP11-35N6.1 - PLASTICITY RELATED GENE 3
201693_s_at	455.64	158.85	0.45	1.30E-02	EGR1 - EARLY GROWTH RESPONSE 1
209948_at	284.37	111.01	0.45	2.00E-03	KCNMB1 - POTASSIUM LARGE CONDUCTANCE CALCIUM-ACTIVATED CHANNEL, SUBFAMILY M, BE...
32094_at	155.26	69.67	0.45	1.00E-03	CHST3 - CARBOHYDRATE (CHONDROITIN 6) SULFOTRANSFERASE 3
219615_s_at	80.10	41.27	0.45	1.40E-02	KCNK5 - POTASSIUM CHANNEL, SUBFAMILY K, MEMBER 5
221004_s_at	139.22	65.23	0.44	1.80E-02	ITM2C - INTEGRAL MEMBRANE PROTEIN 2C
214927_at	576.04	197.88	0.44	1.20E-02	ITGBL1 - INTEGRIN, BETA-LIKE 1 (WITH EGF-LIKE REPEAT DOMAINS)
213900_at	607.01	266.97	0.44	1.30E-02	C9ORF61 - CHROMOSOME 9 OPEN READING FRAME 61
204781_s_at	82.78	38.54	0.44	1.10E-02	TRPC1 - TRANSIENT RECEPTOR POTENTIAL CATION CHANNEL, SUBFAMILY C, MEMBER 1
209047_at	514.63	193.48	0.44	2.00E-03	PDGFD - PLATELET DERIVED GROWTH FACTOR D
209135_at	180.10	72.64	0.44	2.00E-03	PLAGL1 - PLEIOMORPHIC ADENOMA GENE-LIKE 1
208079_s_at	1370.05	423.60	0.44	1.70E-02	MT1E - METALLOTHIONEIN 1E (FUNCTIONAL)
206847_s_at	63.86	25.56	0.44	2.50E-02	HOXA7 - HOMEOBOX A7
202947_s_at	124.07	50.52	0.44	1.10E-02	GYPC - GLYCOPHORIN C (GERBICH BLOOD GROUP)
60474_at	73.71	33.64	0.43	2.00E-03	C20ORF42 - CHROMOSOME 20 OPEN READING FRAME 42
221935_s_at	83.73	29.31	0.43	1.70E-02	NCAM1 - NEURAL CELL ADHESION MOLECULE 1
213620_s_at	150.88	62.16	0.43	2.00E-02	ICAM2 - INTERCELLULAR ADHESION MOLECULE 2
218992_at	1950.90	909.03	0.43	1.90E-02	NFIB - NUCLEAR FACTOR I/B
219935_at	90.06	26.40	0.43	1.50E-02	MAB21L1 - MAB-21-LIKE 1 (C. ELEGANS)
32094_at	68.35	29.26	0.43	1.20E-02	SENP6 - SUMO1/SENTRIN SPECIFIC PEPTIDASE 6
205249_at	334.49	133.31	0.43	2.20E-02	EGR2 - EARLY GROWTH RESPONSE 2 (KROX-20 HOMOLOG, DROSOPHILA)
206766_at	237.55	96.86	0.43	1.00E-03	ITGA10 - INTEGRIN, ALPHA 10
222073_at	399.24	161.67	0.43	1.10E-02	SERPING1 - SERPIN PEPTIDASE INHIBITOR, CLADE G (C1 INHIBITOR), MEMBER 1, (ANGIOED...
202191_s_at	325.70	121.87	0.43	5.00E-03	GAS7 - GROWTH ARREST-SPECIFIC 7
205066_s_at	141.41	53.39	0.43	2.30E-02	TNXB - TENASCIN XB
201719_s_at	549.88	227.67	0.43	2.10E-02	EPB41L2 - ERYTHROCYTE MEMBRANE PROTEIN BAND 4.1-LIKE 2
219498_s_at	78.13	33.71	0.43	6.00E-03	BCL11A - B-CELL CLL/LYMPHOMA 11A (ZINC FINGER PROTEIN)
204457_s_at	497.49	197.68	0.43	2.20E-02	GAS1 - GROWTH ARREST-SPECIFIC 1
209047_at	248.06	96.78	0.43	3.00E-03	AQP1 - AQUAPORIN 1 (COLTON BLOOD GROUP)
204508_s_at	159.59	66.93	0.43	5.00E-03	RGN - REGUCALCIN (SENESCENCE MARKER PROTEIN-30)
201984_s_at	168.06	65.09	0.42	0.00E+00	EGFR - EPIDERMAL GROWTH FACTOR RECEPTOR (ERYTHROBLASTIC LEUKEMIA VIRAL (V-ERB...
213591_at	99.34	38.18	0.42	9.00E-03	ALDH7A1 - ALDEHYDE DEHYDROGENASE 7 FAMILY, MEMBER A1
219497_s_at	121.25	48.77	0.42	1.00E-03	BCL11A - B-CELL CLL/LYMPHOMA 11A (ZINC FINGER PROTEIN)
215380_s_at	113.57	40.96	0.42	1.00E-03	NFASC - NEUROFASCIN HOMOLOG (CHICKEN)
214844_s_at	483.17	191.90	0.41	1.00E-03	ST5 - SUPPRESSION OF TUMORIGENICITY 5
214439_x_at	183.39	78.83	0.41	2.30E-02	PTGER4 - PROSTAGLANDIN E RECEPTOR 4 (SUBTYPE EP4)
222073_at	92.09	31.98	0.41	1.00E-03	COL4A3 - COLLAGEN, TYPE IV, ALPHA 3 (GOODPASTURE ANTIGEN)
205081_at	198.42	76.52	0.41	2.20E-02	SIRPA - PROTEIN TYROSINE PHOSPHATASE, NON-RECEPTOR TYPE SUBSTRATE 1
202274_at	436.13	160.90	0.40	2.00E-02	ACTG2 - ACTIN, ALPHA 2, SMOOTH MUSCLE, AORTA
211712_s_at	232.93	77.74	0.40	1.00E-02	PDGFA - PLATELET-DERIVED GROWTH FACTOR ALPHA POLYPEPTIDE

204793_at	280.34	95.81	0.40	4.00E-03	GPRASP1 - G PROTEIN-COUPLED RECEPTOR ASSOCIATED SORTING PROTEIN 1
208650_s_at	241.07	98.55	0.40	1.70E-02	SAA1 - SERUM AMYLOID A1/ SERUM AMYLOID A2
207016_s_at	147.66	58.32	0.40	6.00E-03	MAPRE2 - MICROTUBULE-ASSOCIATED PROTEIN, RP/EB FAMILY, MEMBER 2
213005_s_at	127.88	46.16	0.40	4.00E-03	MFGE8 - MILK FAT GLOBULE-EGF FACTOR 8 PROTEIN
208399_s_at	118.84	46.96	0.40	1.30E-02	TESC - TESCALCIN
218567_x_at	159.33	57.52	0.40	0.00E+00	SYN2 - SYNAPSIN II
204235_s_at	203.05	75.06	0.40	4.00E-03	GULP1 - GULP, ENGULFMENT ADAPTOR PTB DOMAIN CONTAINING 1
202381_at	215.83	76.29	0.39	1.00E-03	NR3C2 - NUCLEAR RECEPTOR SUBFAMILY 3, GROUP C, MEMBER 2
37577_at	117.88	46.37	0.39	0.00E+00	ARHGAP19 - RHO GTPASE ACTIVATING PROTEIN 19
205117_at	303.09	96.69	0.39	3.00E-03	FGF1 - FIBROBLAST GROWTH FACTOR 1 (ACIDIC)
49452_at	79.91	29.20	0.39	4.00E-03	NR2F1 - NUCLEAR RECEPTOR SUBFAMILY 2, GROUP F, MEMBER 1
202746_at	262.64	102.41	0.39	1.00E-03	ITM2A - INTEGRAL MEMBRANE PROTEIN 2A
200021_at	108.95	41.42	0.39	1.00E-03	SEMA5A - SEMA DOMAIN, SEVEN THROMBOSPONDIN REPEATS
219064_at	134.37	51.36	0.38	2.00E-03	ITIH5 - INTER-ALPHA (GLOBULIN) INHIBITOR H5
202596_at	123.43	47.35	0.38	1.60E-02	TNXB - TENASCIN XB
201540_at	625.92	175.31	0.38	1.20E-02	FHL1 - FOUR AND A HALF LIM DOMAINS 1
207030_s_at	358.56	123.57	0.38	1.00E-03	CSRP2 - CYSTEINE AND GLYCINE-RICH PROTEIN 2
201096_s_at	377.30	126.85	0.38	9.00E-03	MID1 - MIDLINE 1 (OPITZ/BBB SYNDROME)
203323_at	107.05	42.83	0.38	1.50E-02	RUNX3 - RUNT-RELATED TRANSCRIPTION FACTOR 3
209392_at	132.68	48.82	0.38	1.60E-02	TNN - TENASCIN N
206121_at	254.64	97.55	0.38	2.00E-03	MEIS2 - MEIS1, MYELOID ECOTROPIC VIRAL INTEGRATION SITE 1 HOMOLOG 2 (MOUSE)
64900_at	133.52	45.07	0.37	1.00E-03	LPHN2 - LATROPHILIN 2
219498_s_at	448.49	152.83	0.37	3.00E-03	MYH11 - MYOSIN, HEAVY POLYPEPTIDE 11, SMOOTH MUSCLE
213502_x_at	742.55	251.43	0.37	2.10E-02	LOC91316 – 8
209613_s_at	56.02	19.50	0.37	2.10E-02	MAF - V-MAF MUSCULOAPONEUROTIC FIBROSARCOMA ONCOGENE HOMOLOG (AVIAN)
202723_s_at	149.36	46.87	0.37	1.00E-02	FOXO1 - FORKHEAD BOX O1A (RHABDOMYOSARCOMA)
209540_at	202.49	53.08	0.36	2.00E-02	IGF1 - INSULIN-LIKE GROWTH FACTOR 1 (SOMATOMEDIN C)
200621_at	1363.07	474.14	0.36	2.00E-03	CSRP1 - CYSTEINE AND GLYCINE-RICH PROTEIN 1
205752_s_at	199.49	67.13	0.36	0.00E+00	GSTM5 - GLUTATHIONE S-TRANSFERASE M5
210980_s_at	188.81	58.00	0.36	1.00E-03	MPPE1 - METALLOPHOSPHOESTERASE 1
201719_s_at	385.41	134.02	0.36	1.00E-03	TNXB - TENASCIN XB
204422_s_at	1198.52	371.51	0.36	1.00E-03	TXNIP - THIOREDOXIN INTERACTING PROTEIN
204359_at	189.43	60.88	0.35	0.00E+00	FLRT2 - FIBRONECTIN LEUCINE RICH TRANSMEMBRANE PROTEIN 2
204237_at	122.38	44.73	0.35	1.00E-03	GULP1 - GULP, ENGULFMENT ADAPTOR PTB DOMAIN CONTAINING 1
203349_s_at	426.50	118.04	0.35	4.00E-03	ETV5 - ETS VARIANT GENE 5 (ETS-RELATED MOLECULE)
214247_s_at	65.13	22.13	0.35	2.00E-03	SPON1 - SPONDIN 1, EXTRACELLULAR MATRIX PROTEIN
204437_s_at	207.80	77.07	0.34	1.30E-02	FOLR1 - FOLATE RECEPTOR 1 (ADULT)
202274_at	241.56	85.80	0.34	1.00E-03	LY75 - LYMPHOCYTE ANTIGEN 75
209168_at	185.60	63.35	0.34	0.00E+00	GPM6B - GLYCOPROTEIN M6B
215515_at	93.49	29.73	0.34	2.00E-03	KIRREL - KIN OF IRRE LIKE (DROSOPHILA)
218549_s_at	103.59	32.67	0.34	3.00E-03	TRIM9 - TRIPARTITE MOTIF-CONTAINING 9
823_at	445.02	131.75	0.34	5.00E-03	CX3CL1 - CHEMOKINE (C-X3-C MOTIF) LIGAND 1
203481_at	88.28	31.27	0.34	5.00E-03	NA
209270_at	336.21	108.09	0.34	2.50E-02	LAMB3 - LAMININ, BETA 3
209335_at	528.56	158.61	0.33	1.20E-02	DCN - DECORIN
203980_at	508.34	145.90	0.33	3.00E-03	TRIM29 - TRIPARTITE MOTIF-CONTAINING 29
206765_at	207.61	65.37	0.33	1.40E-02	KCNJ2 - POTASSIUM INWARDLY-RECTIFYING CHANNEL, SUBFAMILY J, MEMBER 2
213315_x_at	304.16	97.24	0.33	2.20E-02	SNAI2 - SNAIL HOMOLOG 2 (DROSOPHILA)
213645_at	448.98	124.67	0.33	1.10E-02	TMEM49 - TRANSMEMBRANE PROTEIN 49
204908_s_at	2737.24	833.21	0.33	3.00E-03	MYLK - MYOSIN, LIGHT POLYPEPTIDE KINASE
220751_s_at	308.94	88.10	0.33	2.00E-03	NDN - NECDIN HOMOLOG (MOUSE)
208712_at	50.20	15.18	0.32	1.00E-03	S100B - S100 CALCIUM BINDING PROTEIN, BETA (NEURAL)
201983_s_at	173.88	43.75	0.32	2.00E-03	TFPI - TISSUE FACTOR PATHWAY INHIBITOR
204754_at	145.94	42.82	0.31	1.00E-03	HLF - HEPATIC LEUKEMIA FACTOR
203924_at	47.92	23.36	0.31	2.40E-02	GSTA1 - GLUTATHIONE S-TRANSFERASE A1
201641_at	121.23	24.45	0.31	5.00E-03	PTHLH - PARATHYROID HORMONE-LIKE HORMONE
203881_s_at	533.05	131.25	0.31	1.10E-02	DMD - DYSTROPHIN (MUSCULAR DYSTROPHY, DUCHENNE AND BECKER TYPES)
211712_s_at	408.52	120.93	0.31	0.00E+00	MIA - MELANOMA INHIBITORY ACTIVITY

203755_at	223.60	64.87	0.30	0.00E+00	PTPRZ1 - PROTEIN TYROSINE PHOSPHATASE, RECEPTOR-TYPE, Z POLYPEPTIDE 1
214651_s_at	112.52	32.66	0.30	2.00E-03	HOXA9 - HOMEOBOX A9
213800_at	217.80	62.29	0.30	2.20E-02	CFH - COMPLEMENT FACTOR H
202956_at	56.79	13.74	0.30	4.00E-03	PDLIM3 - PDZ AND LIM DOMAIN 3
200974_at	3587.55	1081.79	0.30	2.00E-03	ACTG2 - ACTIN, ALPHA 2, SMOOTH MUSCLE, AORTA
201983_s_at	253.45	72.56	0.30	2.00E-03	EGFR - EPIDERMAL GROWTH FACTOR RECEPTOR (ERYTHROBLASTIC LEUKEMIA VIRAL (V-ERB...
205348_s_at	170.34	44.55	0.30	6.00E-03	TCEAL2 - TRANSCRIPTION ELONGATION FACTOR A (SII)-LIKE 2
204850_s_at	126.58	30.48	0.30	2.00E-03	DCX - DOUBLECORTEX; LISSENCEPHALY, X-LINKED (DOUBLECORTIN)
203065_s_at	314.82	71.46	0.30	1.00E-03	RUNX1T1 - RUNT-RELATED TRANSCRIPTION FACTOR 1; TRANSLOCATED TO, 1 (CYCLIN D-RELA...
212738_at	219.87	67.31	0.30	2.00E-03	ARHGAP19 - RHO GTPASE ACTIVATING PROTEIN 19
204591_at	515.82	115.35	0.29	2.00E-03	SEMA5A - SEMA DOMAIN, SEVEN THROMBOSPONDIN REPEATS (TYPE 1 AND TYPE 1-LIKE)
219935_at	484.44	109.58	0.29	6.00E-03	ADAMTS5 - ADAM METALLOPEPTIDASE WITH THROMBOSPONDIN TYPE 1 MOTIF, 5 AGGRECANASE
204755_x_at	168.23	48.49	0.29	0.00E+00	HLF - HEPATIC LEUKEMIA FACTOR
211653_x_at	329.64	82.58	0.29	1.40E-02	NTRK2 - NEUROTROPHIC TYROSINE KINASE, RECEPTOR, TYPE 2
212677_s_at	57.01	16.87	0.29	2.10E-02	SCN2A - SODIUM CHANNEL, VOLTAGE-GATED, TYPE II, ALPHA 2
210347_s_at	380.55	63.28	0.28	2.20E-02	MYH11 - MYOSIN, HEAVY POLYPEPTIDE 11, SMOOTH MUSCLE
207016_s_at	144.60	36.55	0.28	4.00E-03	ALDH1A2 - ALDEHYDE DEHYDROGENASE 1 FAMILY, MEMBER A2
202747_s_at	365.69	97.53	0.28	1.20E-02	ITM2A - INTEGRAL MEMBRANE PROTEIN 2A
202022_at	399.88	90.95	0.28	2.00E-03	MATN2 - MATRILIN 2
209613_s_at	57.72	12.68	0.28	1.80E-02	ADH1C - ALCOHOL DEHYDROGENASE 1A (CLASS I), ALPHA POLYPEPTIDE
219497_s_at	1184.07	302.17	0.27	2.00E-03	MYH11 - MYOSIN, HEAVY POLYPEPTIDE 11, SMOOTH MUSCLE
204971_at	880.91	107.38	0.27	2.00E-03	CSTA - CYSTATIN A (STEFIN A)
213001_at	59.69	16.16	0.27	1.00E-02	PAK3 - P21 (CDKN1A)-ACTIVATED KINASE 3
210347_s_at	134.16	34.95	0.27	1.00E-03	BCL11A - B-CELL CLL/LYMPHOMA 11A (ZINC FINGER PROTEIN)
202972_s_at	58.83	15.04	0.27	5.00E-03	TRIM29 - TRIPARTITE MOTIF-CONTAINING 29
209335_at	407.53	90.18	0.26	1.00E-03	SOBP - HYPOTHETICAL PROTEIN FLJ10159
209283_at	418.68	105.19	0.26	5.00E-03	CRYAB - CRYSTALLIN, ALPHA B
209392_at	501.20	145.87	0.26	1.40E-02	ENPP2 - ECTONUCLEOTIDE PYROPHOSPHATASE/PHOSPHODIESTERASE 2 (AUTOTAXIN)
204591_at	104.58	25.25	0.26	8.00E-03	CHL1 - CELL ADHESION MOLECULE WITH HOMOLOGY TO L1CAM (CLOSE HOMOLOG OF L1)
218717_s_at	99.95	30.47	0.26	6.00E-03	LEPREL1 - LEPRECAN-LIKE 1
204850_s_at	242.48	69.54	0.26	5.00E-03	SOX10 - SRY (SEX DETERMINING REGION Y)-BOX 10
218980_at	127.35	29.65	0.26	0.00E+00	FHOD3 - FORMIN HOMOLOGY 2 DOMAIN CONTAINING 3
213900_at	176.53	40.96	0.25	7.00E-03	RELN - REELIN
205666_at	184.97	43.33	0.25	5.00E-03	FMO1 - FLAVIN CONTAINING MONOOXYGENASE 1
216594_x_at	238.65	56.83	0.25	2.00E-02	NTRK2 - NEUROTROPHIC TYROSINE KINASE, RECEPTOR, TYPE 2
213004_at	88.08	23.39	0.25	9.00E-03	PCDH9 - PROTOCADHERIN 9
206121_at	96.30	24.93	0.25	1.70E-02	AMPD1 - ADENOSINE MONOPHOSPHATE DEAMINASE 1 (ISOFORM M)
209570_s_at	167.80	43.40	0.24	7.00E-03	CXORF40B - CHROMOSOME X OPEN READING FRAME 40A
204753_s_at	120.00	32.65	0.24	1.30E-02	HLF - HEPATIC LEUKEMIA FACTOR
213844_at	361.62	93.41	0.24	3.00E-03	HOXA5 - HOMEOBOX A5
205003_at	445.92	71.82	0.24	3.00E-03	SRPX - SUSHI-REPEAT-CONTAINING PROTEIN, X-LINKED
201818_at	172.04	38.65	0.24	0.00E+00	POU2AF1 - POU DOMAIN, CLASS 2, ASSOCIATING FACTOR 1
205824_at	66.48	13.89	0.24	2.00E-03	HSPB2 - HEAT SHOCK 27KDA PROTEIN 2
203980_at	339.60	70.93	0.23	4.00E-03	FABP4 - FATTY ACID BINDING PROTEIN 4, ADIPOCYTE
203687_at	275.13	48.94	0.23	6.00E-03	CSTA - CYSTATIN A (STEFIN A)
220262_s_at	130.64	32.90	0.23	4.00E-03	SPON1 - SPONDIN 1, EXTRACELLULAR MATRIX PROTEIN
209170_s_at	551.32	124.82	0.23	1.30E-02	GPM6B - GLYCOPROTEIN M6B
204422_s_at	51.30	14.13	0.22	5.00E-03	FGF2 - FIBROBLAST GROWTH FACTOR 2 (BASIC)
214974_x_at	82.95	15.08	0.22	1.40E-02	CX3CL1 - CHEMOKINE (C-X3-C MOTIF) LIGAND 1
201098_at	334.80	36.07	0.21	1.50E-02	SFRP1 - SECRETED FRIZZLED-RELATED PROTEIN 1
211719_x_at	101.87	23.26	0.20	1.60E-02	WNT5B - WINGLESS-TYPE MMTV INTEGRATION SITE FAMILY, MEMBER 5B
209169_at	176.45	42.30	0.19	1.10E-02	GPM6B - GLYCOPROTEIN M6B
209167_at	818.85	168.54	0.19	1.00E-03	GPM6B - GLYCOPROTEIN M6B
211726_s_at	751.55	120.70	0.19	0.00E+00	FMO2 - FLAVIN CONTAINING MONOOXYGENASE 2

215946_x_at	1233.44	208.01	0.18	2.30E-02	IGLL3 - SIMILAR TO OMEGA PROTEIN
202965_s_at	231.14	24.60	0.18	1.00E-02	CAPN6 - CALPAIN 6
208399_s_at	147.38	22.74	0.18	2.00E-03	EDN3 - ENDOTHELIN 3
201754_at	1531.75	170.04	0.16	8.00E-03	SFRP1 - SECRETED FRIZZLED-RELATED PROTEIN 1
202966_at	79.30	11.07	0.15	9.00E-03	CAPN6 - CALPAIN 6
203766_s_at	269.85	44.31	0.15	8.00E-03	LMOD1 - LEIOMODIN 1 (SMOOTH MUSCLE)
64900_at	55.14	6.28	0.13	1.00E-03	NPY2R - NEUROPEPTIDE Y RECEPTOR Y2
206121_at	1366.76	179.56	0.13	6.00E-03	OXTR - OXYTOCIN RECEPTOR
204851_s_at	45.01	5.13	0.13	5.00E-03	DCX - DOUBLECORTEX; LISSENCEPHALY, X-LINKED (DOUBLECORTIN)
211935_at	388.09	42.46	0.10	4.00E-03	MME - MEMBRANE METALLO-ENDOPEPTIDASE
209905_at	122.46	16.12	0.10	3.00E-03	HOXA9 - HOMEOBOX A9
210980_s_at	118.53	11.16	0.09	2.10E-02	PKP1 - PLAKOPHILIN 1 (ECTODERMAL DYSPLASIA/SKIN FRAGILITY SYNDROME)
202641_at	288.82	27.07	0.07	2.00E-03	MME - MEMBRANE METALLO-ENDOPEPTIDASE
