# Supplementary material for: Expression of microRNA and their gene targets are dysregulated in preinvasive breast cancer
Source: Breast Cancer Res. 2011 Mar 4;13(2):R24. doi: 10.1186/bcr2839 (PMC3219184; doi:10.1186/bcr2839)
Supplement: Additional file 6 — Expression correlation of microRNA and coordinately expressed predicted targets. [file bcr2839-S6.PDF]

S6. Table-Expression correlation of miRNAs and coordinately expressed predicted targets.

microRNA expression		Gene expression		miRNA:mRNA		
miRNA	Fold Change	Probe ID	Gene Symbol-Gene Name	Fold Change	Expression correlation	p-value
let-7c	0.03	219935_at	ADAMTS5-ADAM metallopeptidase with thrombospondin type 1 motif, 5 (aggrecanase-2)	0.29	0.72	1.82E-03
		213001_at	ANGPTL2-angiopoietin-like 2	0.49	0.59	1.64E-02
		213004_at		0.51	0.37	1.56E-01
		221935_s_at	C3ORF64-chromosome 3 open reading frame 64	0.51	0.56	2.51E-02
		204851_s_at	DCX-doublecortex; lissencephaly, X-linked (doublecortin)	0.13	0.59	1.69E-02
		204850_s_at		0.30	0.49	5.52E-02
		203881_s_at	DMD-dystrophin	0.31	0.55	2.65E-02
		209905_at	HOXA9-homeobox A9	0.10	0.84	5.60E-05
		214651_s_at		0.31	0.82	9.73E-05
		206121_at	MEIS2-Meis1, myeloid ecotropic viral integration site 1 homolog 2 (mouse)	0.38	0.69	2.98E-03
		219696_at	NAB1-NGFI-A binding protein 1 (EGR1 binding protein 1)	0.58	0.73	1.43E-03
miR-10b	0.04	219696_at	BACH2-BTB and CNC homology 1, basic leucine zipper transcription factor 2	0.48	0.14	6.09E-01
		218486_at	KLF11-Kruppel-like factor 11	0.56	-0.02	9.51E-01
		209335_at	SOBP-sine oculis biding protein homolog	0.26	0.47	6.81E-02
miR-125b	0.05	218486_at	FUT4-Fucosyltransferase 4 (alpha (1,3) fucosyltransferase, myeloid-specific)	0.57	0.48	5.84E-02
miR-145	0.03	219696_at	BACH2-BTB and CNC homology 1, basic leucine zipper transcription factor 2	0.48	0.39	6.93E-02
		202723_s_at	FOXO1-forkhead box O1A (rhabdomyosarcoma)	0.37	0.42	6.62E-04
		202724_s_at		0.46	0.57	2.02E-02
miR-17-3p	0.12	221530_s_at	BHLHB3-basic helix-loop-helix domain containing, class B, 3	0.47	0.73	1.37E-03
		205249_at	EGR2-early growth response 2 (Krox-20 homolog, Drosophila)	0.43	0.20	4.57E-01
		204753_s_at	HLF-hepatic leukemia factor	0.24	0.31	2.38E-01
		204755_x_at		0.29	0.38	1.45E-01
		204754_at		0.31	0.32	2.23E-01
		203963_at	RGL1-ral guanine nucleotide dissociation stimulator-like 1	0.53	0.57	2.23E-02
		204422_s_at	TXNIP-thioredoxin interacting protein	0.36	0.36	1.68E-01
		205117_at		0.49	0.25	3.54E-01
miR-181b	5.46	219402_s_at	DERL1-Der1-like domain family, member 1	2.81	0.48	5.75E-02
		200776_s_at	RAB11A-RAB11A, member RAS oncogene family	1.57	0.38	1.47E-01
miR-182	72.35	201096_s_at	ARF4-ADP-ribosylation factor 4	1.85	0.38	1.47E-01
		200021_at	CFL1-cofilin 1 (non-muscle)	2.05	0.27	3.07E-01
		200021_at	INSIG1-insulin induced gene 1	2.13	0.65	6.00E-03
		209770_at	PTP4A1-Protein tyrosine phosphatase type IVA, member 1	1.78	0.57	2.12E-02
miR-183	51.88	213492_at	SERP1-stress-associated endoplasmic reticulum protein 1	1.80	0.41	1.11E-01
miR-195	0.13	219935_at	ADAMTS5-ADAM metallopeptidase with thrombospondin type 1 motif, 5 (aggrecanase-2)	0.29	0.65	6.38E-03
		219696_at	BACH2-BTB and CNC homology 1, basic leucine zipper transcription factor 2	0.48	0.39	1.33E-01
		221530_s_at	BHLHB3-basic helix-loop-helix domain containing, class B, 3	0.47	0.37	1.57E-01
		202966_at	CAPN6-calpain 6	0.15	0.49	5.29E-02
		202965_s_at		0.18	0.43	9.97E-02
		202965_s_at	D4S234E-DNA segment on chromosome 4 (unique) 234 expressed sequence	0.24	0.74	1.16E-03
		209569_x_at		0.27	0.35	1.82E-01
		205348_s_at	DYNC1I1-dynein, cytoplasmic 1, intermediate chain 1	0.56	0.30	2.67E-01
		204422_s_at	FGF2-fibroblast growth factor 2 (basic)	0.22	0.55	2.79E-02
		205498_at	GHR-growth hormone receptor	0.46	0.47	6.93E-02
		205498_at	KCNJ2-potassium inwardly-rectifying channel, subfamily J, member 2	0.33	0.21	4.31E-01
		206765_at	LPHN2-latrophilin 2	0.37	0.75	7.94E-04
		64900_at	PCDH9-Protocadherin 9	0.25	0.50	4.95E-02
		213004_at	RARB-retinoic acid receptor, beta	0.59	0.44	9.01E-02
		219060_at	RASL12-Ras-like, family 12	0.60	0.29	2.73E-01
		213900_at	RELN-Reelin	0.25	0.59	1.59E-02
		203065_s_at	RUNX1T1-runt-related transcription factor 1 (cyclin D-related)	0.30	0.50	4.60E-02
miR-204	0.08	213001_at	ANGPTL2-angiopoietin-like 2	0.49	0.51	4.19E-02
		213004_at		0.51	0.34	1.93E-01
		207016_s_at	MAPRE2-Microtubule-associated protein, RP/EB family, member2	0.40	0.51	4.13E-02
		210347_s_at	BCL11A-B-cell CLL/lymphoma 11A (zinc finger protein)	0.27	0.77	4.74E-04
		219497_s_at		0.42	0.48	5.84E-02
		219498_s_at		0.43	0.43	9.52E-02
		202274_at	NR3C1-nuclear receptor subfamily 3, group C, member 1 (glucocorticoid receptor)	0.46	0.53	3.44E-02
		200974_at		0.58	0.41	1.13E-01
		213004_at	PCDH9-Protocadherin 9	0.25	0.29	2.73E-01
miR-21	20.92	200776_s_at	RAB11A-RAB11A, member RAS oncogene family	1.57	0.61	1.21E-02
miR-7	5.19	213492_at	SERP1-stress-associated endoplasmic reticulum protein 1	1.80	-0.02	7.55E-02
miR-93	160.47	202381_at	ADAM9-ADAM metallopeptidase domain 9 (meltrin gamma)	2.04	0.69	3.08E-03
		219787_s_at	ECT2-epithelial cell transforming sequence 2 oncogene	2.69	0.65	6.78E-03
		212446_s_at	LASS6-LAG1 longevity assurance homolog 6	1.78	0.47	6.60E-02
		221935_s_at	RAD23B-RAD23 homolog B	1.79	0.49	5.59E-02
		221935_s_at	SAR1B-SAR1 gene homolog B	1.95	0.56	2.33E-02
		202723_s_at	YWHAZ-tyrosine 3-monooxygenase/tryptophan 5-monooxygenase activation protein, zeta polypeptide	2.34	0.51	4.54E-02
