# Supplementary material for: Expression of microRNA and their gene targets are dysregulated in preinvasive breast cancer
Source: Breast Cancer Res. 2011 Mar 4;13(2):R24. doi: 10.1186/bcr2839 (PMC3219184; doi:10.1186/bcr2839)
Supplement: Additional file 7 — Enriched gene ontology terms for predicted target genes. [file bcr2839-S7.PDF]

S7. Table. Enriched gene ontology terms for all predicted target genes from the DAVID bioinformatics resource.

GO Term~ Biological Process	# Genes	% Gene List	P-Value	Gene Symbols	Fold Enrichment	FDR
GO:0006355~ regulation of transcription, DNA-dependent	23	31.1%	7.21E-05	RUNX1T1, FOXN3, BHLHB3, HLF, EGR1, LASS6, KLF11, NAB1, NRIP1, CBX7, HOXA9, FOXO1, MEIS2, RAB11A, EZH2, BCL11A, HMGB3, NR3C1, TXNIP, NFIB, RARB, BACH2, EGR2	2.14	0.12
GO:0045449~regulation of transcription	24	32.4%	7.40E-05	RUNX1T1, FOXN3, BHLHB3, HLF, EGR1, LASS6, KLF11, NAB1, NRIP1, CBX7, HOXA9, FOXO1, MEIS2, RAB11A, WWP1, EZH2, BCL11A, NR3C1, HMGB3, TXNIP, NFIB, RARB, BACH2, EGR2	2.09	0.12
GO:0032774~RNA biosynthetic process	23	31.1%	1.01E-04	RUNX1T1, FOXN3, BHLHB3, HLF, EGR1, LASS6, KLF11, NAB1, NRIP1, CBX7, HOXA9, FOXO1, MEIS2, RAB11A, EZH2, BCL11A, HMGB3, NR3C1, TXNIP, NFIB, RARB, BACH2, EGR2	2.11	0.17
GO:0006351~transcription, DNA-dependent	23	31.1%	1.01E-04	RUNX1T1, FOXN3, BHLHB3, HLF, EGR1, LASS6, KLF11, NAB1, NRIP1, CBX7, HOXA9, FOXO1, MEIS2, RAB11A, EZH2, BCL11A, HMGB3, NR3C1, TXNIP, NFIB, RARB, BACH2, EGR2	2.11	0.17
GO:0006350~transcription	24	32.4%	1.02E-04	RUNX1T1, FOXN3, BHLHB3, HLF, EGR1, LASS6, KLF11, NAB1, NRIP1, CBX7, HOXA9, FOXO1, MEIS2, RAB11A, WWP1, EZH2, BCL11A, NR3C1, HMGB3, TXNIP, NFIB, RARB, BACH2, EGR2,	2.05	0.17
GO:0019219~regulation of nucleobase, nucleoside, nucleotide and nucleic acid metabolic process	24	32.4%	1.40E-04	RUNX1T1, FOXN3, BHLHB3, HLF, EGR1, LASS6, KLF11, NAB1, NRIP1, CBX7, HOXA9, FOXO1, MEIS2, RAB11A, WWP1, EZH2, BCL11A, NR3C1, HMGB3, TXNIP, NFIB, RARB, BACH2, EGR2,	2.02	0.23
GO:0031323~regulation of cellular metabolic process	26	35.1%	1.76E-04	RUNX1T1, HLF, LASS6, KLF11, NAB1, NRIP1, WWP1, FGF2, TXNIP, RARB, EGR2, BACH2, FOXN3, BHLHB3, EGR1, CBX7, FOXO1, HOXA9, MEIS2, RAB11A, EZH2, BCL11A, HMGB3, NR3C1, CCND1, NFIB,	1.91	0.29
GO:0010468~regulation of gene expression	24	32.4%	1.89E-04	RUNX1T1, FOXN3, BHLHB3, HLF, EGR1, LASS6, KLF11, NAB1, NRIP1, CBX7, HOXA9, FOXO1, MEIS2, RAB11A, WWP1, EZH2, BCL11A, NR3C1, HMGB3, TXNIP, NFIB, RARB, BACH2, EGR2,	1.99	0.31
GO:0019222~regulation of metabolic process	26	35.1%	3.03E-04	RUNX1T1, HLF, LASS6, KLF11, NAB1, NRIP1, WWP1, FGF2, TXNIP, RARB, EGR2, BACH2, FOXN3, BHLHB3, EGR1, CBX7, FOXO1, HOXA9, MEIS2, RAB11A, EZH2, BCL11A, HMGB3, NR3C1, CCND1, NFIB,	1.86	0.50
GO:0016070~RNA metabolic process	23	31.1%	7.92E-04	RUNX1T1, FOXN3, BHLHB3, HLF, EGR1, LASS6, KLF11, NAB1, NRIP1, CBX7, HOXA9, FOXO1, MEIS2, RAB11A, EZH2, BCL11A, HMGB3, NR3C1, TXNIP, NFIB, RARB, BACH2, EGR2,	1.88	1.30
GO:0010467~gene expression	24	32.4%	2.45E-03	RUNX1T1, FOXN3, BHLHB3, HLF, EGR1, LASS6, KLF11, NAB1, NRIP1, CBX7, HOXA9, FOXO1, MEIS2, RAB11A, WWP1, EZH2, BCL11A, NR3C1, HMGB3, TXNIP, NFIB, RARB, BACH2, EGR2,	1.72	3.97
GO:0043283~biopolymer metabolic process	35	47.3%	2.78E-03	SERP1, RUNX1T1, HLF, PTP4A1, TRIB1, NMT2, LASS6, KLF11, NAB1, NRIP1, RELN, WWP1, FGF2, TXNIP, FUT4, RARB, EGR2, BACH2, FOXN3, BHLHB3, EGR1, PRKD1, CBX7, DERL1, HOXA9, FOXO1, RAD23B, MEIS2, RAB11A, EZH2, BCL11A, HMGB3, NR3C1, CCND1, NFIB	1.46	4.49
GO:0007264~small GTPase mediated signal transduction	10	13.5%	1.13E-02	RGL1, RELN, RAB11A, ECT2, FGF2, ARL8B, ARF4, RASL12, SAR1B, CFL1	2.37	17.10
GO:0007154~cell communication	32	43.2%	1.74E-02	DOK4, D4S234E, RASL12, SAR1B, NAB1, NRIP1, RELN, WWP1, FGF2, SLC20A1, RARB, SNCA, EGR2, CD164, RGL1, LPHN2, ARL8B, ARF4, DCX, MAPRE2, NCAM1, PRKD1, CFL1, DERL1, FOXO1, YWHAZ, RAB11A, ADAM9, ECT2, ANGPTL2, NR3C1, CCND1	1.37	25.19
GO:0016481~negative regulation of transcription	7	9.5%	2.08E-02	FOXN3, MEIS2, WWP1, EGR1, KLF11, NAB1, NRIP1	2.81	29.24
GO:0010001~glial cell differentiation	4	5.4%	2.24E-02	RELN, FGF2, NAB1, EGR2	5.22	31.16

GO:0045892~negative regulation of transcription, DNA-dependent	5	6.8%	2.60E-02	FOXN3, MEIS2, EGR1, KLF11, NRIP1	3.73	35.20
GO:0007242~intracellular signaling cascade	18	24.3%	2.88E-02	RGL1, DOK4, ARL8B, ARF4, DCX, RASL12, PRKD1, SAR1B, CFL1, NRIP1, DERL1, RELN, RAB11A, ADAM9, ECT2, FGF2, SLC20A1, CCND1	1.59	38.23
GO:0031324~negative regulation of cellular metabolic process	7	9.5%	3.09E-02	FOXN3, MEIS2, WWP1, EGR1, KLF11, NAB1, NRIP1	2.61	40.45
GO:0045934~negative regulation of nucleobase, nucleoside, nucleotide and nucleic acid metabolic process	7	9.5%	3.09E-02	FOXN3, MEIS2, WWP1, EGR1, KLF11, NAB1, NRIP1	2.61	40.45
GO:0043170~macromolecule metabolic process	40	54.1%	3.25E-02	SERP1, RUNX1T1, HLF, PTP4A1, TRIB1, NMT2, LASS6, KLF11, NAB1, NRIP1, RELN, WWP1, FGF2, DPP3, TXNIP, FUT4, CAPN6, RARB, BACH2, EGR2, FOXN3, BHLHB3, EGR1, RIMS3, PRKD1, CBX7, DERL1, HOXA9, FOXO1, RAD23B, MEIS2, ADAMTS5, RAB11A, ADAM9, EZH2, BCL11A, NR3C1, HMGB3, CCND1, NFIB	1.24	42.00
GO:0007165~signal transduction	28	37.8%	4.24E-02	DOK4, D4S234E, RASL12, SAR1B, NRIP1, RELN, WWP1, FGF2, SLC20A1, RARB, CD164, RGL1, LPHN2, ARL8B, ARF4, DCX, MAPRE2, PRKD1, CFL1, DERL1, FOXO1, YWHAZ, RAB11A, ADAM9, ECT2, ANGPTL2, NR3C1, CCND1	1.34	51.04
GO:0000122~negative regulation of transcription from RNA polymerase II promoter	4	5.4%	4.84E-02	MEIS2, EGR1, KLF11, NRIP1	4.18	55.87
GO:0042063~gliogenesis	4	5.4%	4.84E-02	RELN, FGF2, NAB1, EGR2	4.18	55.87

GO Term~ Molecular Function	# Genes	% Gene List	P-Value	Gene Symbols	Fold Enrichment	FDR
GO:0003700~transcription factor activity	15	20.3%	8.03E-04	FOXN3, RUNX1T1, BHLHB3, HLF, EGR1, LASS6, KLF11, HOXA9, FOXO1, MEIS2, NR3C1, NFIB, RARB, EGR2, BACH2	2.40	1.17
GO:0030528~transcription regulator activity	17	23.0%	1.23E-03	RUNX1T1, FOXN3, BHLHB3, HLF, EGR1, LASS6, KLF11, NAB1, NRIP1, HOXA9, FOXO1, MEIS2, NR3C1, NFIB, RARB, EGR2, BACH2	2.15	1.78
GO:0003677~DNA binding	18	24.3%	2.76E-03	RUNX1T1, FOXN3, BHLHB3, HLF, EGR1, LASS6, KLF11, HOXA9, FOXO1, RAD23B, MEIS2, EZH2, HMGB3, NR3C1, NFIB, RARB, EGR2, BACH2	1.96	3.96
GO:0043565~sequence-specific DNA binding	9	12.2%	1.56E-02	HOXA9, FOXO1, FOXN3, MEIS2, HLF, NR3C1, LASS6, RARB, BACH2	2.45	20.52
GO:0003676~nucleic acid binding	19	25.7%	2.43E-02	RUNX1T1, FOXN3, BHLHB3, HLF, EGR1, LASS6, KLF11, HOXA9, FOXO1, RAD23B, MEIS2, EZH2, BCL11A, HMGB3, NR3C1, NFIB, RARB, EGR2, BACH2	1.59	30.22
GO:0016564~transcription repressor activity	4	5.4%	7.54E-02	FOXN3, MEIS2, NAB1, NRIP1	3.63	68.29

Note: The entire DCIS vs. CN differentially expressed gene list was used as the gene population background (420 genes). C3orf64 did not fall into any of these annotation/ontology categories
