# Supplementary material for: Expression of microRNA and their gene targets are dysregulated in preinvasive breast cancer
Source: Breast Cancer Res. 2011 Mar 4;13(2):R24. doi: 10.1186/bcr2839 (PMC3219184; doi:10.1186/bcr2839)
Supplement: Additional file 8 — Cancer-specific functional annotation and pathway analysis of predicted target genes. [file bcr2839-S8.PDF]

**S8. Table-Cancer specific functional annotation and pathway analysis of all predicted target genes.**

| Gene ID | Description | KEGG Pathway | Pubmed ID:Reference in Function |
|---|---|---|---|
| ADAM9 | adam-metallopeptidase domain 9 | | ***12767059**:Expression of ADAM-9 mRNA and protein in human breast cancer.* ***14997207**:Cytoplasmic ADAM9 over-expression is associated with poor differentiation in ductal adenocarcinoma* **15205330**:ADAM9 over-expression enhances cell adhesion and invasion of non-small cell lung cancer cells via modulation of other adhesion molecules and changes in sensitivity to growth factors, thereby promoting metastatic capacity to the brain., **17704059**:the ADAM-9 adhesive domain plays a role in regulating the motility of cells by interaction with beta1 integrins and modulates MMP synthesis **18061337:**ADAM9 is over-expressed in prostate cancer cases and is an independent prognostic marker of PSA relapse-free survival following radical prostatectomy. |
| ADAMTS5 | adam metallopeptidase with thrombospondin type 1 motif, 5 | | **15599946**:negative effect of TGFbeta1 on ADAMTS-1, -5, -9, and -15 coupled with increases in their inhibitor, TIMP-3 may aid the accumulation of versican in the stromal compartment of the prostate in BPH and prostate cancer **16003758:**ADAMTS4 and 5 are up-regulated on proliferating glioblastoma cells, and these proteases may contribute to their invasive potential, |
| ANGPTL2 | angiopoietin-like 2 | | **18593905:**epigenetic silencing by hypermethylation of the ANGPTL2 promoter leads to a loss of ANGPTL2 function, which may be a factor in the carcinogenesis of ovarian cancer in a stage-dependent manner |
| ARF4 | adp-ribosylation factor 4 | **hsa05110:**Cholera Infection | **19041174:**ARF4 participates in the regulation of glioblastoma apoptosis through the inhibition of stress-mediated apoptotic signals |
| ARL8B | adp-ribosylation factor-like 8b | | **15331635:**Results suggest that the novel GTPases Gie1 and Gie2 associate with microtubules, and might be involved in chromosome segregation. |
| BCL11A | b-cell lymphoma 11a | | **16871282:**essential functional role of this repressor of transcription in primary mediastinal B-cell lymphoma |
| BACH2 | btb and cnc homology 1, basic leucine zipper transcription factor 2 | | **11746976:**BACH2 is transcriptionally regulated by the BCR/ABL oncogene 16832351:Upregulation of BACH2 is associated with ovarian cancer **17044046:**down-regulation of the BACH2 gene through the interaction with centromeric heterochromatin would take part in leukeomogenesis of BCR-ABL positive lymphoid leukemia. |
| BHLHB3 (DEC2) | basic helix-loop-helix domain containing, class b, 3 | **hsa04710:**Circadian rhythm, | **12354771:**DEC1 and DEC2 may play a crucial role in the adaptation to hypoxia **12397359:**Dec1 and Dec2 are regulators of the mammalian molecular clock, and form a fifth clock-gene family. **12624110:**DEC1-mediated repression on the expression of DEC2 **18223678**:role of the BHLHB3 protein as a tumor suppressor for lung cancer. |
| CAPN6 | calpain 6 | | **18657900:**results indicated that calpain 6 supports tumorigenesis by inhibiting apoptosis and facilitating angiogenesis. |
| CBX7 | chromobox homolog 7 | | **14647293:**controls cellular lifespan through regulation of both the p16(Ink4a)/Rb and the Arf/p53 pathways **15897876:**CBX7 represses melanoma, p16, inhibits CDK4 and p14Arf expression in normal and tumor-derived prostate cells, affecting their growth **17374722:**CBX7 is a chromobox protein causally linked to cancer development **18701502:**Loss of the CBX7 gene expression correlates with a highly malignant phenotype in thyroid cancer **18984978:**Downregulation of CBX7 is associated with urothelial tumor progression., |

| | | | |
|---|---|---|---|
| CD164 | cd164 molecule, sialomucin | **hsa04142**:lysosome | **16859559:**CD164 may participate in the localization of prostate cancer cells to the marrow and is further evidence that tumor metastasis and hematopoietic stem cell trafficking may involve similar processes |
| CCND1 | cyclin d1 | **hsa04110:**Cell cycle **hsa04115:**p53 signaling pathway **hsa04310:**Wnt signaling pathway **hsa04510:**Focal adhesion **hsa04630:**Jak-STAT signaling pathway **hsa05210:**Colorectal cancer **hsa05212:**Pancreatic cancer **hsa05213**:Endometrial cancer **hsa05214:**Glioma, **hsa05215**:Prostate cancer **hsa05216:**Thyroid cancer **hsa05218:**Melanoma **hsa05219:**Bladder cancer **hsa05220:**Chronic myeloid leukemia **hsa05221:**Acute myeloid leukemia **hsa05222:**Small cell lung cancer **hsa05223:**Non-small cell lung cancer | ***11923474:**cyclin D1 may be a target gene for prolactin in normal lobuloalveolar development, as well as in the development and/or progression of mammary cancer.* ***11986316:**expression affected by estrogen receptors alpha and beta* ***12007188:**Overexpression of cyclin D1 is significantly correlated with increased chromosomal instability in patients with breast cancer prevalence.* ***12376514:**early use of oral contraceptives may be associated with subset of mammary tumors that over-express cyclin D1,* ***12379776:**Marked intratumoral heterogeneity of c-myc and this but not of c-erbB2 amplification in breast cancer,* ***12825853:**Cyclin D1 could be indirectly induced by ErbB signaling through p21. ER-mediated up-regulation of cyclin D1 seems to be a possible mechanism of maintaining cell proliferation in DCIS in case of EGFR- and HER-2/neu-negativity.* ***12917338:**The cyclin D1 gene is over-expressed in human breast cancers and is required for oncogene-induced tumorigenesis,* ***14612904:**cyclin D1, besides growth pattern, is a prognostic marker for local recurrence in DCIS,* *15282324*:Data show that CCND1 promoter activation by *estrogens in human breast cancer cells is mediated by recruitment of a c-Jun/c-Fos/estrogen receptor alpha/progesterone receptor complex to the tetradecanoyl phorbol acetate-responsive element of the gene.* ***16723714:**Cav-1-deficient mammary acini displayed increased ER-alpha levels and enhanced sensitivity toward estrogen-stimulated growth, with specific up-regulation of cyclin D1* |
| CFL1 | cofilin 1 (non-muscle) | **hsa04360:**Axon guidance **hsa04810:**Regulation of actin cytoskeleton, **h_CCR3Pathway:**CCR3 signaling in Eosinophils **h_rac1Pathway:**Rac 1 cell motility signaling pathway **h_rhoPathway:**Rho cell motility signaling pathway | **17583572:**destrin is a significant regulator of various processes important for invasive phenotype of human colon cancer Isreco1 cells whereas cofilin-1 may be involved in only a subset of them, **18952063:**These results strongly support the importance of cofilin in tight-junction opening, suggesting cofilin as a target for tight-junction permeability regulation in epithelial cells. **18987670:**Plexin C1, a receptor for semaphorin 7a, inactivates cofilin and is a potential tumor suppressor for melanoma progression. |
| COL2A1 | collagen, type ii, alpha 1c | **hsa01430:**Cell Communication **hsa04510:**Focal adhesion **hsa04512:**ECM-receptor interaction | |
| DERL1 | der1-like domain family, member 1 | **hsa05014:**Amyotrophic lateral sclerosis (ALS), | ***18205950:**derlin-1 over-expression in breast cancer, together with its function in relieving ER stress-induced apoptosis, suggests that regulation of the ER stress response pathway may be critical in the development and progression of breast cancer.* **18927294:**Overexpression of DERL1 is associated with neoplasms |
| DOK4 | docking protein 4 | | **17443497:**IRS-5 (DOK4) is significantly up-regulated in 90% of examined clear cell RCCs. Studies on this gene has shown that it is regulated through chromatin remodeling in kidney cells, |

| | | | |
|---|---|---|---|
| DOK4 | docking protein 4 | | **17443497:**IRS-5 (DOK4) is significantly up-regulated in 90% of examined clear cell RCCs. Studies on this gene has shown that it is regulated through chromatin remodeling in kidney cells,<br>**19073520:**Epigenetic regulation of DOK4 expression is associated with non-small-cell lung cancer., |
| DPP3 | dipeptidyl-peptidase 3 | | **14529681:**In malignant neoplasms of the ovary DPP III activity increased with growing histologic grade. |
| ECT2 | epithelial cell transforming sequence 2 oncogene | | **15254234:**ECT2 regulates the polarity complex Par6/Par3/PKCzeta and possibly plays a role in epithelial cell polarity<br>**16778203:**ECT2 knockdown triggers cell cycle arrest in G1 |
| EGR1 | early growth response 1 | **hsa04514:**Cell adhesion molecules (CAMs)<br>**hsa05020:** Prion diseases | **12553019:**Egr-1 has a significant role in carcinogenesis and in cancer progression, especially metastasis.<br>**12706485:**These results suggest that induction of egr-1 may be needed to regulate genes involved in DNA repair, cell survival, and apoptosis.<br>***15999367:**The EGR1 gene appeared to be deleted in ER-negative human breast carcinomas. Egr-1 may contribute to the pathogenesis of ER-negative breast carcinomas versus ER-positive breast carcinomas.*<br>**16093249:**EGR1 regulates heparanase transcription in tumor cells and importantly, can have a repressive or activating role depending on the tumor type,<br>***18204200**:These results suggest that Egr-1 may be an important breast cancer marker and that an as yet uncharacterized pathway involved in Egr-1 and gelsolin expression exists which leads to breast cancer cell development.* |
| EGR2 | early growth response 2 | | **14596916:**a possible molecular mechanism to account for down-regulation of EGR2 in tumor cells |
| EZH2 | enhancer of zeste homolog 2 | | **14500907:**functional role of EZH2 in cancer cell invasion and breast cancer progression<br>***14965441**:deregulated expression of EZH2 is associated with loss of differentiation and development of poorly differentiated breast cancer in humans*<br>**15208672:**Activated p53 suppresses EZH2 expression, suggesting a further role for p53 in epigenetic regulation and in the maintenance of genetic stability<br>***16855786**:We summarize the current knowledge on the function of EZH2 in cancer, with special focus on breast cancer, and propose a link between EZH2, the homologous recombination pathway of DNA repair, and breast tumorigenesis.,*<br>***17018586:Review**. EZH2 is up-regulated in ductal carcinoma in situ, atypical ductal hyperplasia, and even morphologically normal breast epithelial cells from women who have an increased risk of breast cancer. EZH2 may promote neoplastic conversion.*<br>***17453341:**EZH2 regulates the transcription of estrogen-responsive genes through association with REA, an estrogen receptor corepressor*<br>***18269588:**EZH2 expression was associated with decreased survival of patients with basal-like phenotype of breast cancer.*<br>**19008416:**study proposes that the genomic loss of miR-101 in cancer leads to over-expression of EZH2 and concomitant dysregulation of epigenetic pathways, resulting in cancer progression,<br>***19079346:**EZH2 is important in ER-negative breast cancer growth in vivo and in vitro, and BRCA1 is required for the proliferative effects of EZH2*<br>***9099573:**EZH2 over-expression is associated with TP53 mutation in breast cancer.* |
| FGF2 | fibroblast growth factor 2 (basic) | **hsa04010:**MAPK signaling pathway,<br>**hsa04810:**Regulation of actin cytoskeleton,<br>**hsa05218:**Melanoma | |

| | | | |
|---|---|---|---|
| FOXN3 | forkhead box n3 | | **16102918**:Data suggest that CHES1 recruits Ski-interacting protein (SKIP) to repress genes important for tumorigenesis and the response to cancer treatments |
| FOXO1 | forkhead box o1a (rhabdomyosarcoma) | **hsa04910:**Insulin signaling pathway **hsa05215**:Prostate cancer **hsa05200:** Pathways in cancer | |
| FUT4 | fucosyltransferase 4 (alpha (1,3) fucosyltransferase, myeloid-specific) | **hsa00602:**Glycosphingolipid biosynthesis - neo-lactoseries **hsa01031:**Glycan structures - biosynthesis 2 | |
| GHR | growth hormone receptor | **hsa04060**:Cytokine-cytokine receptor interaction, **hsa04080:**Neuroactive ligand-receptor interaction, **hsa04630:**Jak-STAT signaling pathway, | ***17287408:****increased risk of breast cancer with higher GHBP,* |
| HLF | hepatic leukemia factor | | |
| HOXA9 | homeobox a9 | | **11830496**:the fusion gene NUP98-HOXA9 is an important gene in myeloid leukemogenesis |
| INSIG1 | insulin induced gene 1 | | **12242342**:INSIG-1 plays a role in regulating cholesterol concentration in human cells, |
| KLF11 | kruppel-like factor 11 | | **15300592**:novel mechanism in TGF-beta-regulated gene expression: KLF11 potentiates Smad-signaling activity in normal epithelial cells through termination of the negative feedback loop imposed by Smad7 |
| LASS6 | lag1 homolog, ceramide synthetase 6 | | |
| MAPRE2 | microtubule-associated protein rp/eb protein 2 | | **16007168:**results support the novel hypothesis that EB1 over-expression may play a role in the development of esophageal squamous cell carcinoma by affecting APC function and activating the beta-catenin/TCF pathway, **16763565**:Impaired EB1 or APC function generates lesions invisible to the spindle checkpoint and thereby promotes low levels of chromosomal loss (CIN) expected to fuel aneuploidy and possibly tumorigenesis. |
| MEIS2 | meis homeobox 2 | | |
| NCAM1 | neural cell adhesion molecule 1 | **hsa04514:**Cell adhesion molecules (CAMs) **hsa05020:** Prion diseases | **16211277:**NCAM is associated not only with a cell-to-cell adhesion mechanism, but also with tumorigenesis, including growth, development and perineural invasion in human salivary gland tumors, |
| NMT2 | N-myristoyltransferase 2 | | **16530191:**a higher expression of NMT2 in 84.6% colorectal carcinoma compared to normal tissues (84.6%) and NMT2 interacts with caspase-3 **16364039**:involved in progression and regulation of telomerase **11731439**:inappropriate protein NH(2)-terminal myristoylation appears to play a role in carcinogenesis; induction ofNMT2 may play a central role in dioxin carcinogenicity **16123142**:NMT1 and NMT2 have partially overlapping functions;NMT1 is critical for tumor cell proliferation |

| | | | |
|---|---|---|---|
| NR3C1 | nuclear receptor subfamily 3, group c, member 1 (glucocorticoid receptor) | **hsa04080:**Neuroactive ligand-receptor interaction | ***15590693:****Glucocorticoid receptor-induced MPK-1 expression inhibits paclitaxel-associated MAPK activation and contributes to breast cancer cell survival*<br>***17512111:****progesterone and glucocorticoid, but not estrogen receptor mRNA are altered in breast cancer stroma,*<br>**17952860:**Breast cancer progression is associated with the accumulation of glucocorticoid receptor in the cytoplasm of tumoral cells and the decrease of COX-2 expression., |
| NRIP1 (RIP140) | nuclear receptor interacting protein 1 | | **12773562:**RIP140 has a role in binding to nuclear receptors, as well as additional functions mediated by the formation and intranuclear relocalization of a repressive protein complex,<br>***15632153:****RIP140 has a regulatory role in mediating anti-estrogenic effects of RA in estrogen-dependent breast cancer cells*,<br>***16439465:****RIP140 differentially regulates ERR activity depending on the target sequence on the promoters.*<br>**17880687:**RIP140 discriminates among different classes of retinoic acid target genes. RIP140 limits RA signaling & tumor-cell differentiation. RIP140 silencing sensitizes embryonal carcinoma cells to low doses of RA.<br>***19401155****:The results presented here suggested the cooperative transcriptional regulation of estrogen signaling by FHL1 and RIP140.* |
| PRKD1 | | | **11978539:**REVIEW: multifunctional role of PKD in processes such as cell proliferation, apoptosis, immune cell regulation, tumor cell invasion and regulation of Golgi vesicle fission<br>**19329994:**Data identify protein kinase D1 as a key regulator of the SSH1L-cofilin signaling pathway and directed cell migration in tumor cells., |
| PTP4A1 | protein tyrosine phosphatase type 4, member 1 | | **12235145:**PRL-1 function is regulated in a cell cycle-dependent manner and implicate PRL-1 in regulating progression through mitosis, possibly by modulating spindle dynamics<br>**18997816:**the new oncogenic p53 target, PRL-1, may contribute to tumor development by the down-regulation of p53 by a negative feedback mechanism., |
| RAB11A | rab11a, member ras oncogene family | **hsa04144:** endocytosis | **15188492:**Rab11a, RPL7, and RPL28 showed moderate levels of differential expression in esophageal squamous cell carcinoma, |
| RAD23B | rad23b homolog | **hsa03420:**nucleotide excision repair | **15550378**:the human nucleotide excision repair gene, hHR23B, is epigenetically silenced in interleukin-6-responsive multiple myeloma KAS-6/1 cells,<br>***16712842:****determined that hHR23A and hHR23B could be co-purified with unique proteolytic and stress-responsive factors from human breast cancer tissues, indicating that they have unique functions in vivo,* |
| RARB | retinoic acid receptor, beta | **hsa05222:**Small cell lung cancer<br>**hsa05223:**Non-small cell lung cancer<br>**hsa05200:** Pathways in cancer | ***11980632****:Endogenous reactivation of the RARbeta2 tumor suppressor gene epigenetically silenced in breast cancer.*<br>***12579317:****ATRA increased RARbeta2 mRNA in non-metastatic breast cancer cells. The same treatment of metastatic cells resulted in an increase in RARbeta4 & a decrease in RARbeta2 mRNA. RARbeta4 may contribute to metastatic properties of breast cancer cell lines.*<br>***14601057:****hypermethylated in invasive and in in situ lobular breast cancer*<br>***18483325:****Methylation of RARB was significantly positively correlated with breast cytological atypia, increasing between ages 35 and 45*<br>***19258476:****The combination of RARB M4, INK4a/ARF, PRB, and HIN-1 CpG island promoter methylation may predict non-BRCA1/2-associated mammary carcinogenesis and tumor progression.* |
| RELN | reelin | **hsa01430:**Cell Communication,<br>**hsa04510:**Focal adhesion,<br>**hsa04512:**ECM-receptor interaction, | **11880184**:Upregulation of reelin is associated with tumorigenesis of esophagus, |
| RUNX1T1 | runt-related transcription factor 1; translocated to, 1 | **hsa05221:**Acute myeloid leukemia<br>**hsa05200:** Pathways | |

| | | | |
|---|---|---|---|
| | (cyclin d-related) | in cancer | |
| SDC1 | syndecan 1 | **hsa04512:**ECM-receptor interaction **hsa04514:**Cell adhesion molecules (CAMs), | **12879463**:High syndecan-1 expression in breast carcinoma is related to an aggressive phenotype, **12920224:**The increased stromal syndecan-1 expression, coupled with its loss from the surface of carcinoma cells, may contribute to tumor cell invasion and the development of metastases, **15383330**:Altered matrix-dependent signaling due to increased levels of cell surface syndecan-1 may lead to epithelial cell invasion during early stages of tumorigenesis., **15459490:**Concomitant expression of syndecan-1 in both epithelium and stroma may be a predictor of unfavorable prognosis in breast cancer, and in contrast with previous studies, loss of epithelial syndecan-1 was associated with a more favorable prognosis, **15743035**:syndecan-1 has a role in progression of invasive breast carcinomas through the remodeling of breast cancer tissue via interaction with other extracellular matrix components, **16247452**:stromal fibroblast-derived Sdc1 stimulates breast carcinoma growth and angiogenesis in vivo, **16636895**:Syndecan-1 and syndecan-4 may have roles in progression of breast carcinoma, **18542065**:loss of syndecan-1 epithelial expression was of strong prognostic value in breast carcinomas, **18657535**:Our findings thus suggest that a previously unknown link between integrin alpha2beta1 and syndecan-1 is important in regulating cell adhesion to collagen and in triggering integrin downstream signaling., **19010933**:Membrane type 1 matrix metalloproteinase-mediated stromal syndecan-1 shedding stimulates breast carcinoma cell proliferation., **19126645**:Proteolytic conversion of Sdc1 from a membrane-bound into a soluble molecule marks a switch from a proliferative to an invasive phenotype, with implications for breast cancer diagnostics and potential glycosaminoglycan-based therapies. |
| SNCA | synuclein, alpha (non a4 component of amyloid precursor) | **hsa01510**:Neurodegenerative Diseases **hsa05010:**Alzheimer's disease **hsa05020:**Parkinson's disease | |
| TRIB1 | tribbles homolog 1 | | **15299019**:Tribbles controls both the extent and the specificity of MAPK kinase activation of MAPK |
| TXNIP | | | **12821938**:VDUP1 is a novel antitumor gene which forms a transcriptional repressor complex **18202760**:D-allose, a simple monosaccharide, may act to cause TXNIP induction and p27kip1 protein stabilization in tumor cells **18301748**:TXNIP is directly repressed by FOXO1a, modulating the cellular response to oxidative stress and affecting life span |
| WWP1 | ww domain containing e3 ubiquitin protein ligase 1 | **hsa04120:**Ubiquitin mediated proteolysis **hsa05050:**Dentatorubropallidoluysian atrophy (DRPLA) **hsa04144**: endocytosis | **15221015:**WWP1 negatively regulates TGF-beta signaling in cooperation with Smad7. **16223724**:KLF5 is a target of the E3 ubiquitin ligase WWP1 for proteolysis in epithelial cells **16924229:**these findings identify the first instance of an ubiquitin ligase that causes stabilization of p53 while inactivating its transcriptional activities., **17016436:**WWP1 over-expression is a common mechanism involved in the inactivation of TGFbeta function in human cancer. *17330240:genomic aberrations of WWP1 may contribute to the pathogenesis of breast cancer* **18724389:**WWP1 may promote cell proliferation and survival partially through suppressing RNF11-mediated ErbB2 and EGFR down-regulation in human cancer cells., |

| | | | |
|---|---|---|---|
| WWP1 | ww domain containing e3 ubiquitin protein ligase 1 | **hsa04120:**Ubiquitin mediated proteolysis<br>**hsa05050:**Dentatorubropallidoluysian atrophy (DRPLA)<br>**hsa04144**: endocytosis | **15221015:**WWP1 negatively regulates TGF-beta signaling in cooperation with Smad7.<br>**16223724**:KLF5 is a target of the E3 ubiquitin ligase WWP1 for proteolysis in epithelial cells<br>**16924229:**these findings identify the first instance of an ubiquitin ligase that causes stabilization of p53 while inactivating its transcriptional activities.,<br>**17016436:**WWP1 over-expression is a common mechanism involved in the inactivation of TGFbeta function in human cancer.<br>***17330240***:*genomic aberrations of WWP1 may contribute to the pathogenesis of breast cancer*<br>**18724389:**WWP1 may promote cell proliferation and survival partially through suppressing RNF11-mediated ErbB2 and EGFR down-regulation in human cancer cells.,<br>**18806757:**WWP1 may have a context-dependent role in regulating cell survival through targeting different p63 proteins for degradation.,<br>**19047365:**WWP1 ubiquitinated and caused the degradation of HER4 but not of EGFR, HER2, or HER3.<br>***19267401:****Overexpression of WWP1 is associated with the estrogen receptor and insulin-like growth factor receptor 1 in breast carcinoma* |
